# Identification of a Cryptic Pocket in Methionine Aminopeptidase-II Using Adaptive Bandit Molecular Dynamics Simulations and Markov State Models

**DOI:** 10.1021/acsomega.4c02516

**Published:** 2024-06-18

**Authors:** Rubina, Syed Tarique Moin, Shozeb Haider

**Affiliations:** † Third World Center for Science and Technology, H.E.J. Research Institute of Chemistry, International Center for Chemical and Biological Sciences, University of Karachi, Karachi 75270, Pakistan; ‡ UCL School of Pharmacy, University College London, London WC1N 1AX, U.K.; § UCL Centre for Advanced Research Computing, University College London, London WC1H 9RN, U.K.

## Abstract

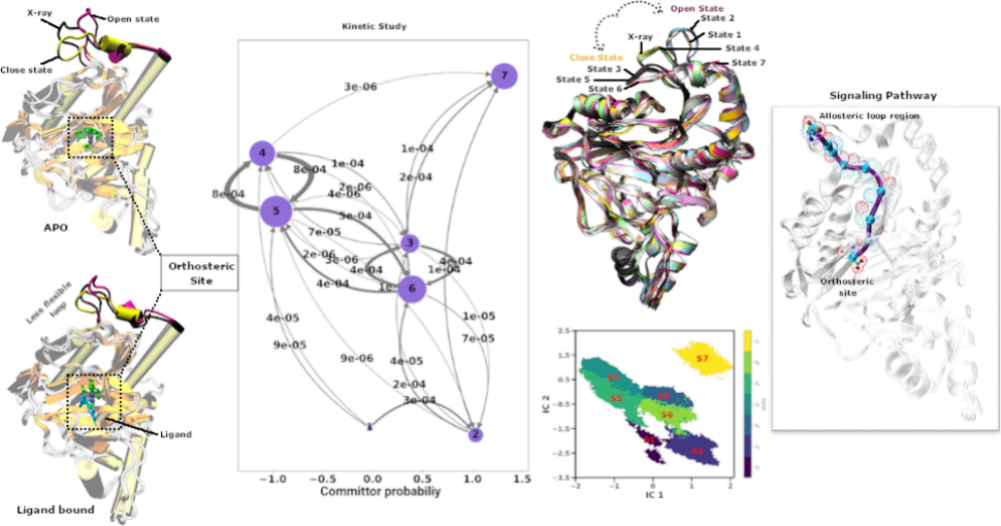

Methionine aminopeptidase-II (MetAP-II) is a metalloprotease, primarily responsible for the cotranslational removal of the N-terminal initiator methionine from the nascent polypeptide chain during protein synthesis. MetAP-II has been implicated in angiogenesis and endothelial cell proliferation and is therefore considered a validated target for cancer therapeutics. However, there is no effective drug available against MetAP-II. In this study, we employ Adaptive Bandit molecular dynamics simulations to investigate the structural dynamics of the apo and ligand-bound MetAP-II. Our results focus on the dynamic behavior of the disordered loop that is not resolved in most of the crystal structures. Further analysis of the conformational flexibility of the disordered loop reveals a hidden cryptic pocket that is predicted to be potentially druggable. The network analysis indicates that the disordered loop region has a direct signaling route to the active site. These findings highlight a new way to target MetAP-II by designing inhibitors for the allosteric site within this disordered loop region.

## Introduction

**Figure 1:**
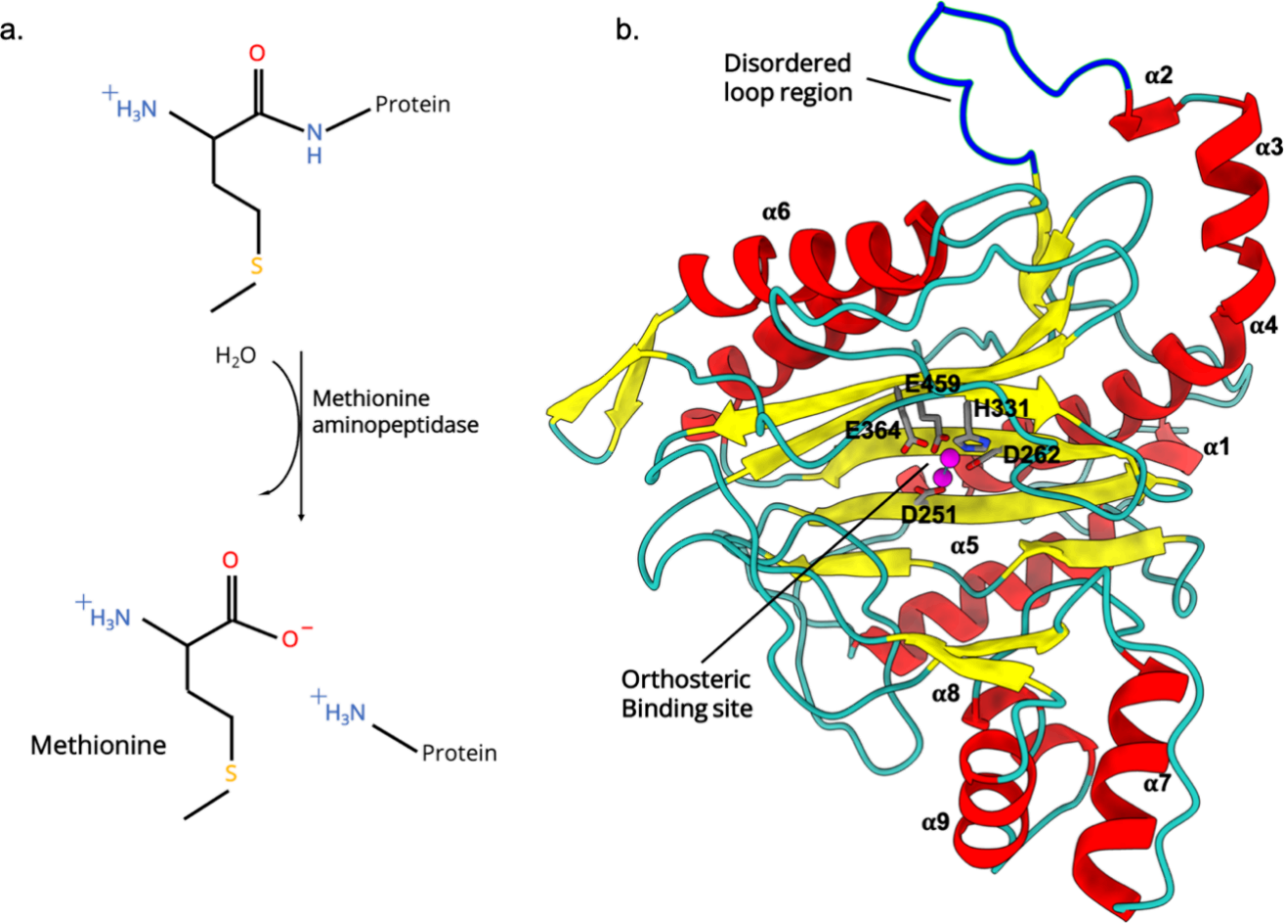
(a) General reaction for the removal of initiator methionine from the newly synthesized polypeptide chain. (b) Structure of MetAP-II. Cartoon representation of MetAP-II (PDB entry 1YW9), showing α-helix (red) and β-sheet (yellow). The disordered loop region that is absent in most of the crystal structure is represented in blue. The orthosteric site of MetAP-II is involved in removal of methionine, containing metal ions (magenta) and its coordinating amino acid residues are represented as sticks (cpk).

Methionine aminopeptidases (MetAPs) are metalloenzymes that remove the first methionine at the N-terminal end in the nascent polypeptide chain (Figure 1a).^1^ This cleavage process is also necessary for further modification of the N-terminal region of the polypeptide chains^2^ such as N-terminal acetylation^3^ and myristoylation.^4^ Aberrations in removal of the terminal methionine results in the polypeptide chain to form inactive protein products.^5^

Human MetAPs (hMetAP) have two isoforms – type I and type II. Both are the cytosolic, monomeric metalloprotease^6^ and are involved in angiogenesis.^7^ hMetAP-I is involved in the G2/M phase transition of the cell cycle,^8^ whereas hMetAP-II participates in G1/S phase.^9^ About 22% of the MetAP-II sequence matches that of the MetAP-I. The type II isozyme can be easily distinguished from the type I by an additional helical subdomain of 64 amino acid (residues 381–444)^10^ located in the catalytic domain.^11^ There is also a connector region (residues 90–139) in hMetAP-I that is absent in hMetAP-II. Additionally, there is an N-terminal domain in hMetAP-II that matches 48% sequence homology with the connector region in the type I enzyme. In both isoforms, the metal binding residues are conserved whereas almost all of the residues that form the methionine-binding pocket are different.^12^

hMetAp-II is composed of 478 amino acids and is split into two domains, the N-terminal domain and the catalytic C-terminal domain (residues 166 to 478). The N-terminal domain has 165 polyacidic and polybasic amino acid residues, which differentiates hMetAp-II from other types of MetAPs (EcMetAp-I and PfMetAp-II). The residues from 1 to 109 and 138–153 are highly disordered.^13^ The N-terminal domain is not essential for amino peptidase activity, but it plays a crucial role in regulating the global protein synthesis and the cell cycle by controlling the phosphorylation levels of eIF2 and ERK1/2.^14^ The disordered N-terminal domain is absent in all of the crystal structures available in the protein data bank. In contrast, the disordered loop (residues 138–153) is also absent in most of the crystal structures. However, in those structures where it has been resolved, the disordered loop always adopts a semiclosed conformation.

The MetAPs catalytic C-terminal domain adopts a novel pita bread fold.^15^ In the deep cleft of the β-sheet of the catalytic C-terminal domain, there are five conserved metal binding residues namely D251, D262, H331, E364, and E459, which coordinate the metal ions and are actively involved in the protease activity.^2^ Three catalytic reaction mechanisms for the cleavage of methionine by MetAP-II have been proposed based on the involvement of one or two of its metal ions.^1,16,17^ However, the exact catalytic reaction mechanism of MetAP-II is still unclear. Nevertheless, it is considered as enzyme catalysis that involves a nucleophilic substitution reaction.^18^ It is interesting to note that the cleavage of the methionine residue is only possible in the condition when penultimate amino acids (P1′) of the growing chain during protein synthesis are small such as alanine, cysteine, glycine, proline, serine, threonine, or valine.^19^

MetAPs have been shown to lose their protease activity in the presence of EDTA (ethylene diamine tetra-acetic acid), thus suggesting that these are metal-dependent enzymes.^20^ However, there are ambiguities in the role of metal ions under physiological conditions. Numerous studies have been reported based on the activity of MetAPs in the presence of different metal ions, including Co(II) and Zn(II) as metal cofactors for *E. coli* MetAP-Ia,^16,21^ the role of Ni(II), Co(II), Mn(II), and Zn(II) in yeast MetAP-Ib,^15^ Co(II) in Yeast MetAP-IIa,^22^ while Mn(II) and Co(II) are considered as cofactors for MetAP-IIb in humans.^23^ A study based on a selective inhibition of MetAP-II bound to various metal ions demonstrated that Mn (II) ions are the most probable cofactor for hMetAP-II.^24^

MetAP-II plays an important role in tissue repairing, angiogenesis, cancer, endothelial cell proliferation, protein synthesis, and their degradation.^13^ Its high expression has been reported in different types of cancers, including mesothelioma,^25^ lymphomas,^26^ colorectal adenocarcinoma,^27^ hepatoma,^28^ and neuroblastoma.^29^ MetAP-II was first identified as the promising target of the antiangiogenic compounds, for instance, fumagillin and ovalicin.^30^ Inhibition of MetAP-II induces G1 cell cycle arrest, which stops the growth and multiplication of tumor cells.^31^

The focus of MetAP-II inhibitors, including fumagillin analogs, is to irreversibly inhibit the enzyme by making a covalent bond with the H231 using its spiro-epoxide moiety.^30,32^ Several other selective and potent inhibitors based on fumagillin analogs such as TNP-470,^33^ CKD732,^34^ and PPI-2458^32^ have been identified to inhibit MetAP-II leading to the cessation of endothelial cell proliferation.^31^ Other reversible inhibitors of hMetAP-II have also gained much attention, which include bengamides,^35^ anthranilic acid sulfonamides,^36^ 2-hydroxy-3-aminoamides,^37^ and triazole analogs.^38^ These reversible inhibitors affect tumor progression and endothelial cell proliferation demonstrated under *in vitro* and also *in vivo* conditions.^31^ Recently, a new scaffold, cyclic hydroxyl malonic acid (tartronic) diamide, has been introduced for the selective inhibition of MetAP-II; and among these, M8891, an orally active reversible inhibitor, has gained much attention since it has reached clinical trials.^39^ However, there are no FDA-approved drugs against MetAP-II.

In spite of these advances, the dynamic behavior of the resulting enzyme–inhibitor complexes has not been evaluated at the atomistic level yet. Here, we employ Adaptive Bandit sampling MD simulations and carry out Markov state model (MSM) analysis to study the conformational dynamics of hMetAP-II.^40^ Importantly, the results reported here identify a dynamic loop in hMetAP-II that acts as a lid to a previously unidentified cavity. Such cryptic pockets can be potentially exploited in the allosteric modulation of the enzyme. The current study represents a step beyond our current knowledge concerning hMetAP-II research. Previously reported studies have primarily focused on the catalytic site without considering the disordered loop region, with no attention given to its functional implications. Thus, in this study, we not only present new knowledge about the conformational dynamics of hMetAP-II but also identify a novel cryptic pocket with plausible therapeutic meaning. By filling the gap that exists between the structural and functional significance, our findings provide a deeper insight into the conformational diversity in the apo- and ligand-bound MetAP-II, which could help to design novel MetAP-II inhibitors for newly identified cryptic pocket.

## Materials and Methods

### Atomistic Model of MetAP-II and Ligand Parametrization

The MetAP-II structure was obtained from the protein data bank (PDB ID: 1YW9),^41^ which contains a ligand and two manganese (Mn) ions in its active site. There is no crystal structure of the Mn ion containing apo MetAP-II. The apo structure was prepared by removing the ligand from the complex. Additionally, the apo MetAP-II comprises a hydroxyl group that bridges the metal ions.^42^ Each metal is also coordinated to water molecules.^43^ Therefore, the hydroxyl group and water molecules were added to the active site to build apo MetAP-II for simulations. The AMBER ff14sb force field was used to describe the protein structure, whereas the Mn parameters were obtained from the literature.^44^

The crystal structure of MetAP-II in complex with a cyclic tartronic diamide ligand (PDB ID: 6QED)^39^ was deemed unacceptable for use because of a number of missing residues. Therefore, to prepare the ligand complex, the structure of the ligand complex was superimposed onto the apo structure (Cα RMSD 0.29 Å). Subsequently, the coordinates of the ligand were extracted and positioned in the 1YW9 structure. Further, the retrieved ligand structure was modified *in situ* (within the active site) to obtain the M8891 inhibitor used in this study with the help of GaussView.^45^ B3LYP functional^46^ and 6-31G** basis sets were utilized to optimize ligand by applying constraints optimization. The electrostatic potential map for the ligand was calculated using the Hartree–Fock level of theory (HF/6-31G**), and charges were obtained using the restrained electrostatic potential (RESP) scheme.^47,48^ The bonding parameters for ligands were taken from the generalized AMBER force field (GAFF) method.^48^

### Force Field Parametrization of the Mn-Containing Active Site

Both systems (apo and ligand-bound MetAP-II) were prepared using the AMBER ff14sb force field.^48^ The metals in the protein were described as the nonbonded parameters in which electrostatic interaction is computed in terms of Coulomb’s law while the van der Waals is based on Lennard-Jones potential.^49^ Nonbonded van der Waals parameters, epsilon (ε), and sigma (σ) for Mn (II) were assigned as 0.03 kcal/mol and 1.45 Å as adopted from the work by Babu and Lim.^44^

### Adaptive Bandit Sampling Molecular Dynamics Simulations

The initial systems (apo and ligand-bound MetAP-II) were prepared using the PlayMolecule web application (www.playmolecule.org). The pH was set to 7.4. In the case of the ligand-bound MetAP-II, the mol2 file containing the charges for the ligand was provided as an input. The ProteinPrepare module then carries out p*K*_a_ calculations by assigning appropriate charges and optimizes hydrogen bond network.^50^ The output file from ProteinPrepare is then input to the xLEaP module of AmberTools20,^51^ which was used to generate the topology and coordinates of the systems employing the Amber ff14sb force field.^48^ The systems were neutralized by the addition of counterions with 0.15 M Na^+^ and Cl^–^ ions and solvated in a cubic TIP3P water box^52^ whose edges were set to a maximum distance of 12 Å from the closest solute atom.^53^ The systems were initially minimized by using 3000 steps of steepest descent and equilibrated for 5 ns in an NPT ensemble at 1 atm. The temperature was steadily increased to 300 K with a time step of 4 fs by using rigid bonds and a 9 Å cutoff for particle mesh Ewald summation for long-range electrostatics. The protein backbone was restrained during the equilibration. The Berendsen barostat controlled the pressure, while the velocities were based on the Boltzmann distribution. The production step was run by employing the Adaptive Bandit algorithm with default parameters.^40^ Numerous short molecular dynamics simulations were carried out using the ACEMD engine.^50,54^ Each simulation was run in the NVT ensemble using a Langevin thermostat with 0.1 ps damping and a hydrogen mass repartitioning scheme that permitted a 4 fs time step. The MSM-based Adaptive Bandit algorithm utilizes the MetricSelfDistance function to build and respawn further simulations. A total of 400 and 340 trajectories for apo and ligand-bound MetAP-II were run, respectively. Each trajectory was run for 50 ns (500 frames) and saved every 0.1 ns, thereby sampling a total of 20 μs (apo) and 17 μs (ligand-bound).

### Analysis of the MD Trajectories

The simulation data was analyzed using PyTraj^55^ and MDAnalysis.^56^ The simulation trajectories were visualized in VMD.^57^ The structural figures were generated using Chimera,^58^ VMD,^57^ Protein Imager,^59^ and Jmol.^60^ Additionally, CONAN tools^61^ were used to analyze 2000 frames from the entire trajectory to compute average total contacts formed between the heavy atoms of the protein. The interactions were calculated with a cutoff distance of 5 Å.

### Network Analysis

The graph theory was used to investigate the residual interaction networks where each residue in a protein act as a node in the residue interaction network (RIN).^62^ If there is an interaction between the two residues, then an edge between two nodes exists. These edges are weighted according to residue-pair correlation:
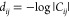
where *dij* is the distance between two contacting nodes *i* and *j*. Pair-wise correlation is represented by *C_ij_*, which generates a graph in which strongly correlated residues are separated by a short distance. In the graph network theory approach, the allosteric pathway between two residues is described by the shortest path between their respective nodes.

The systems were generated by representing each residue as a single Cα node. The two nodes were determined to be in contact if they are within a cutoff distance of 5 Å for more than 75% of the simulation time. The interdependence between nodes is weighted by correlation and is represented as a connecting edge.

The DynOmics^64^ tool uses a network model consisting of nodes representing amino acid residues and connecting edges, denoting the strength of correlations among the residues. The stable structure of apo was uploaded to the elastic network model web of DynOmics. This tool was used in this study for the identification of intramolecular communication and the functional site based on sensor and effector residues. Sensors and effectors are the entities that are recognized by the changes in their responses to structural perturbations. Among them, the sensors (residue *i*) are characterized by having a sensitive response to perturbations and the effector (residue *j*) are distinguished by their effectiveness in relaying information or perturbations to other sites.

Network analysis including betweenness centrality (BC) was evaluated by MDTASK.^62^ The BC plot explores the flow of information across nodes in a network. In the BC plot, the residues with high BC values are involved in communication within the protein.^63^

Trajectories of both systems were reduced to Cα atoms. The calc_network.py script has been used to calculate the BC, whereas the compare_networks.py script was used to compare the networks of the apo and ligand-bound MetAP-II. The number of shortest paths through a vertex is used to characterize its betweenness. The node *x* of the BC is
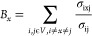
where the total number of shortest paths between *i* and *j* that pass through *x* is defined as σ*_ixj_*. The total number of shortest paths between *i* and *j* is σ*_ij_*. In the BC plot, the residues having high BC values were determined by defining a *z* score in
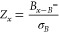
where the average BC value is represented as *B* and the standard deviation by σ*B*. In the BC plot, the residues with *Z_x_* value greater than 2.5 were considered to have high BC.

### Markov State Model Analysis

The PyEMMA program was used to build the Markov State Models (MSM) for the apo, ligand-bound, and combined trajectories of MetAP-II.^65^ First, the most critical step for building an MSM is the selection of features. The backbone torsion angle has previously been reported to be one of the main features for capturing the slow conformational dynamics of the enzyme.^66^ In our case, this feature is sufficient to build an MSM for ligand-bound MetAP-II but is insufficient for the apo enzyme. Since we aim to compare the conformational dynamics of apo and ligand-bound MetAP-II, we need to identify identical features using which we can build our MSMs for both systems. Thus, for the selection of the best common features for our simulated systems, the VAMP-2 score method was applied,^67^ and eight features were explored (Figure S1). From the VAMP-2 score plot, two high-ranked features were selected, which included the distance of the ligand/hydroxyl with all the amino acid residues of MetAP-II and the chi 1 angle of the side chain of the disordered loop region (residues 138–153). To build MSM for the apo and ligand-bound MetAP-II, the selected features mentioned above and the torsion angles of the backbone along with the chi 1 angle of the side chain of the active site (residues F110, P111, G113, A121, H122, D142, D153, A155, L219, N220, H222, I229, H230, T234, E255, F257, H273, M275, A305, L338, Q348, and E350) were included.

The featured trajectories yielded 2332 dimensional data for the apo protein and 3321-dimensional data for ligand-bound protein, which were further projected onto the top three principal components. The reduced dimensional data was clustered using *k*-means clustering into 50 microstates to define the trajectory as a series of transitions between the discrete states. The MSM can then be generated from this discrete trajectory using a suitable lag time because the state generation and transition matrix depends on the choice of lag time.^65^ A lag time of 3 ns has been selected in our study from the converged implied time scale plot. Validation of these generated models was carried out using the implied time scale (ITS) plots and Chapman–Kolmogorov (CK) test implemented in PyEMMA.^65^ The validated MSM model was further used for understanding the intermediate states and kinetics of transition states. Further, the clusters were grouped into the five metastable macrostates based on the kinetic similarity using the Perron cluster–cluster analysis (PCCA+) algorithm.^68^ Finally, the net flux pathways between each metastable state were calculated using the transition path theory function with a predetermined lag time in such a way that model follows the Markovian behavior.^69^ The eigenvectors produced by the eigen decomposition of the transition matrix represent slow dynamics in the systems. Analysis of the obtained macrostates carried out in terms of root-mean-square fluctuation and MDpocket tool.^70^ This tool has been used to explore pockets (cryptic sites) in each macrostate and calculate the volume of cavities.

The interconversion between different conformational states was carried out by performing MSM on the combined trajectories of apo and ligand-bound MetAP-II, after eliminating all hydrogen atoms and removing the ligand. The backbone dihedral angle of the entire protein, the chi 1 angle of the side chain of the active site residues, and the disordered loop region were selected features to build MSM. Time-lagged independent component analysis (TICA) was used to reduce the dimensionality of the simulated data. 100 clusters were generated at a lag time of 3 ns. Finally, a total of seven metastable states were identified.

### Analysis of the Metastable States

The ensemble conformations (1000 frames from each metastable state) obtained from MSM analysis of the combined trajectories were analyzed with MDpocket to identify the pocket volume in each open, intermediate, and closed conformations of MetAP-II. The output result was visualized by PyMOL software (www.pymol.org). Druggability of the binding hotspot was estimated using the FTMap algorithm,^71^ which identified the region of the surface of protein where the ligand binds. The optimal path for signal communication between the identified cryptic pocket and the active site of MetAP-II was evaluated by selecting the residue (Q141) at the tip of the disordered loop and the residue within the active site (E364) that was responsible for enzyme activity. Pathway analysis was carried out using the Weighted Implementation of Suboptimal Pathways method WISP.^72^ In this signaling pathway analysis, the covariance matrix was calculated by defining the center of mass of residues using the keyword RESIDUE_COM, as nodes in the pathway.

## Results and Discussion

The catalytic activity of MetAP-II is affected by a new class of orthosteric inhibitors that have a cyclic tartronic diamide scaffold introduced as the next-generation reversible, selective, and potent inhibitors against MetAP-II.^39^ We have taken the potent inhibitor (M8891) from this class, which is currently in clinical trials.^73^ This inhibitor strongly binds to the orthosteric binding pocket of MetAP-II and remains stable throughout the course of the simulation. The bridging hydroxyl group in the apo MetAP-II initiates the enzymatic activity via a nucleophilic substitution reaction during the catalytic cleavage of methionine from the polypeptide chain. The nucleophilicity of the bridging hydroxyl group increases after the proton is donated to the E364.^1^ In the ligand-bound MetAP-II, the hydroxyl group of the inhibitor replaces the bridging hydroxyl group and makes a strong hydrogen bond with the E364 which is required for the inhibition of enzymatic activity.^1^ The amide oxygen coordinates to the manganese ion (Mn_A_) and the lactam oxygen interacts with the other manganese ion (Mn_B_) as reported in the crystal structures.^39^ The interactions between M8891 and MetAP-II revealed a number of representative interactions including hydrophobic, hydrogen bonding, and π–π stacking of the amino acid residues (Figure S2). The interaction of the inhibitor with E364 blocks the residue from participating in the nucleophilic reaction, thus resulting in the inhibition of the catalytic activity. The amino acid residue L413 in the apo state is involved in an intramolecular interaction with W419 that is affected after the ligand has hydrophobic interactions with L413.

### Structural Dynamics of MetAP-II

The time evolution Cα root-mean-square deviation (RMSD) of both systems (apo and ligand-bound) was computed to obtain preliminary information about the stability of the system. The RMSD analysis (Figure S3) reflects the conformational stability of the protein yielding a comparable value of 1.61 ± 0.21 and 1.63 ± 0.14 Å for the backbone atoms in the apo and the ligand-bound MetAP-II, respectively.

The compactness and structural flexibility of both systems were assessed by calculating the radius of gyration and the averaged root-mean-square fluctuation for the Cα atom of each residue (Figure S4). The resulting plot of the radius of gyration suggested the comparable compactness of the backbone residues of both systems. The RMSF analysis has been performed after removing the roto-translational motion of the residues to characterize per residual fluctuation of the ligand-bound in comparison to its apo MetAP-II. The terminal residues and disordered loop region (residues 138–153) were suggested to be slightly more flexible in the apo state, which upon ligand binding became rigid. The RMSF can also be used to compare the RMSF data with the β-factors obtained from X-ray crystallography and NMR measurements.^55^ These results highlight that the disordered loop region, which exhibits high fluctuations, also possesses high β-factor in experiments (Figure S4).

### Communication within Systems and Allosteric Site Identification

**Figure 2:**
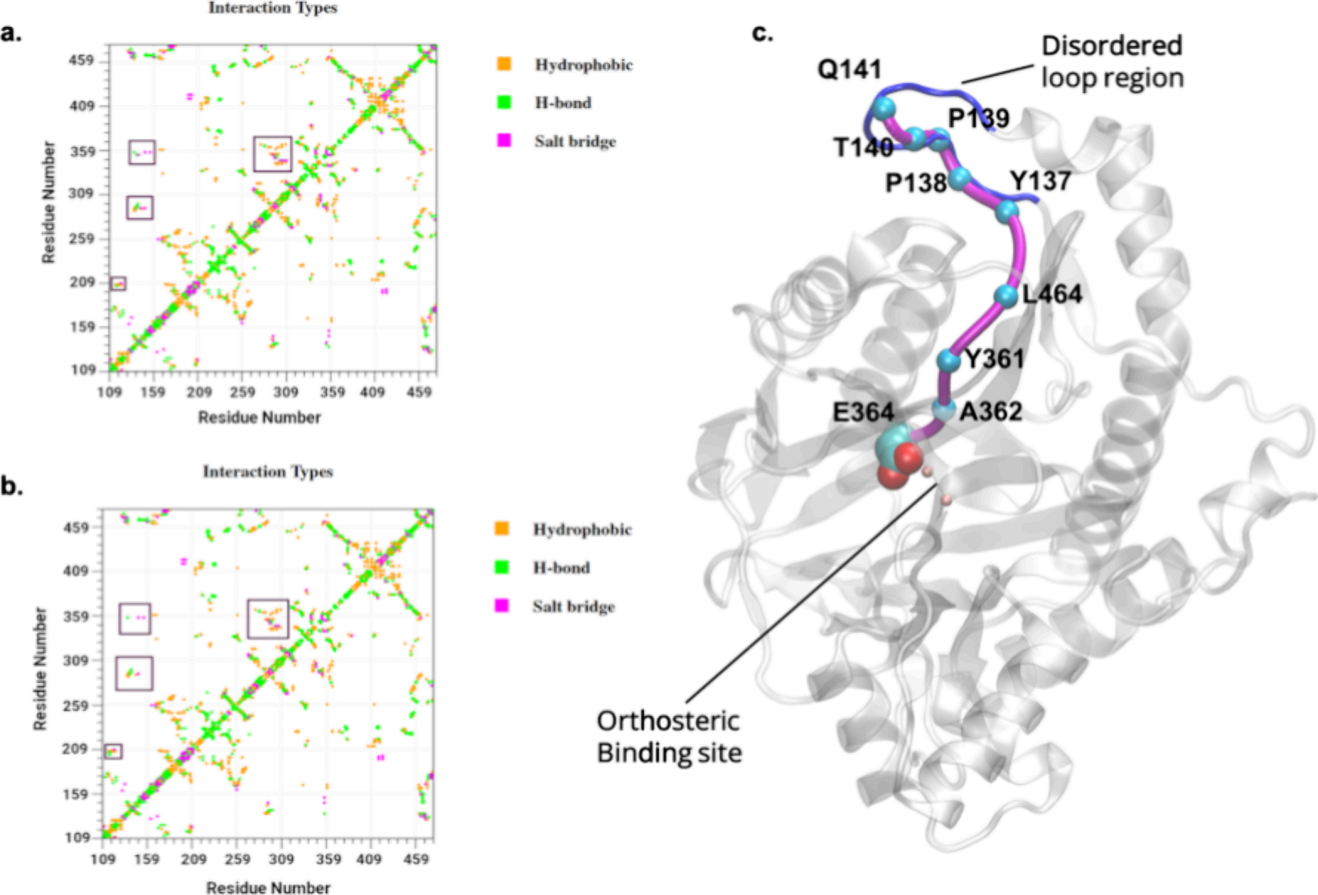
2D interaction plot in (a) apo and (b) ligand-bound MetAP-II. Purple square represents residues that show interaction in apo but not in ligand-bound. (c) Signaling pathway from the identified cryptic pocket (disordered loop region) to the orthosteric site of MetAP-II.

Intramolecular interactions, including hydrophobic, H-bonding, and π–π stacking, that provide stability to the ligand in the binding pocket were evaluated by plotting a 2D intramolecular interaction plot (Figure 2). The inter-residual interactions that were present in the apo MetAP-II state were altered in the ligand-bound state. Additionally, some new interactions were formed that stabilized the protein–ligand complex (Table S1). For instance, the main chain of L413 forms strong intramolecular interaction with the main chain of W419 in the apo state, which was lost after the fluorine atom of the ligand interacts with the main chain of L413 (Figure S2). Moreover, it is interesting to note that the interactions in the disordered loop region are altered after ligand binding in the orthosteric pocket (Figure 2). It must be emphasized that this disordered loop region is 29.8 Å from the orthosteric ligand-binding site.

**Figure 3:**
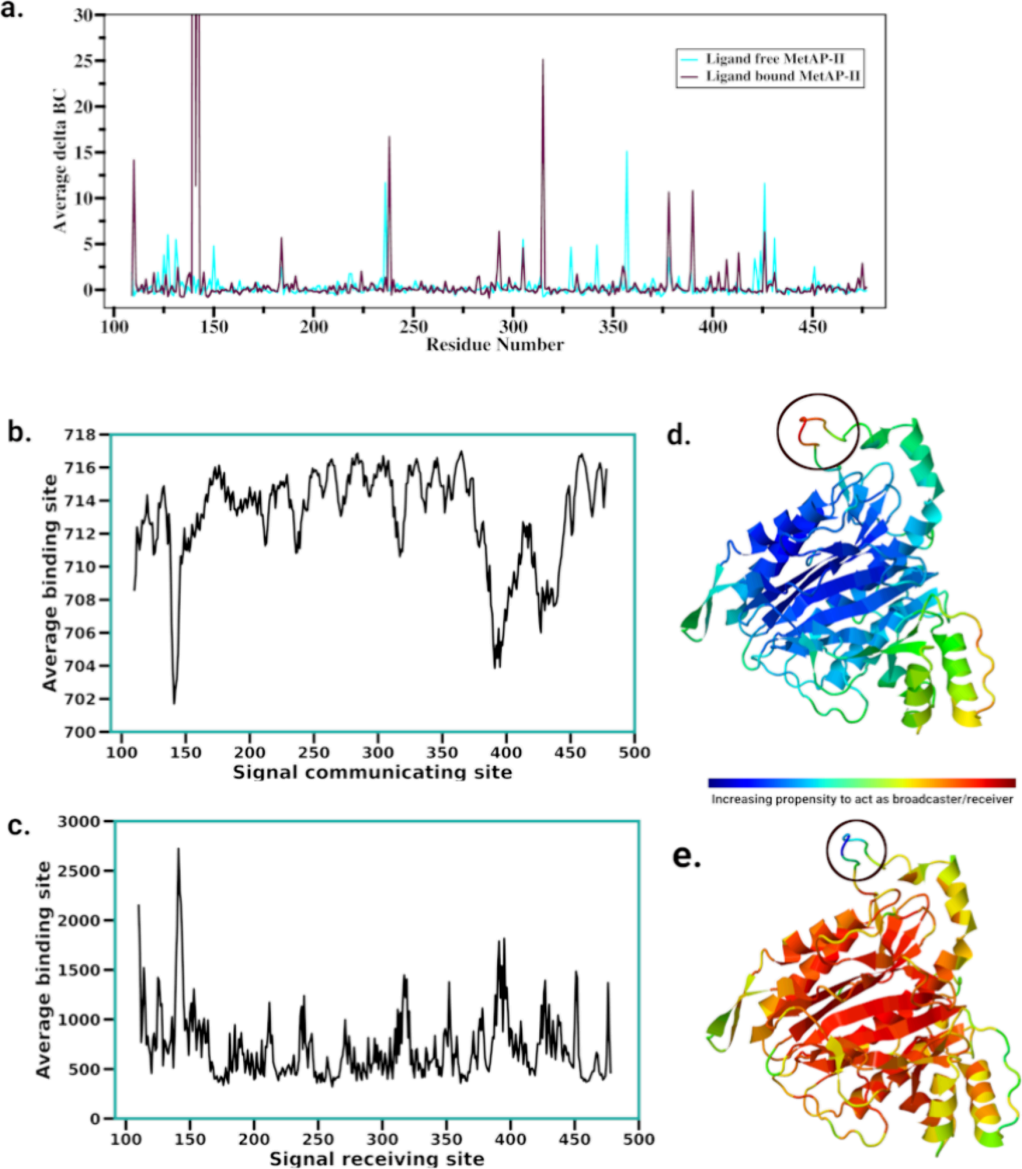
Betweenness centrality in apo and ligand-bound MetAP-II. (a) In the BC plot, a large ΔBC value indicates strong intramolecular interaction. Residues from the disordered loop region in the range of 141–143 have a high BC value in ligand-bound MetAP-II, indicating strong intramolecular communication of the active site residues with this loop region. Signal communication properties: (b) Average communicating response of all residues; low values denote the propensity of residues to send signals. (c) Average receiving responses of all residues; high values indicate the propensity of residues to receive signals. (d) Sites of signal communicating residues; the red color region has a high propensity to send signals, and (e) sites of signal receiving efficiency of each residue; the red color indicates a strong signal receiving tendency.

The role of each residue as a signal sensor and receiver during allosteric signal communication was identified (Figure 3). The disordered loop and residues in the range 375–450 displayed a high propensity of signal transmission, while the active site residues demonstrated more signal-receiving strength (Figure 3b,c). Based on this finding, we propose that the disordered loop region and the residues 375–450 form a potential allosteric site due to their high propensity of sending signals to the orthosteric active site.

To assess if there was a direct communication between the disordered loop (newly identified cryptic pocket) and the orthosteric site, we carried out betweenness centrality analysis and the inter-residual communication in the protein. In the apo state, the residues belonging to the disordered loop region range (residues 141–143) yielded BC values of 1.45, 0.0, and 1.07, thus demonstrating almost no communication with other residues of the protein. However, in the ligand-bound state, these residues were involved in strong inter-residual communication yielding BC values of 310, 11, and 84.8 (Table S2).

Due to the flexibility of the disordered loop region, the cryptic site was also explored with the help of F*pocket* tools in the apo MetAP-II. The identified pocket could be functionally important and act as a conventional site for allosteric ligand binding.^74^ Based on the F*pocket* analysis, 27 binding pockets were identified along with the orthosteric site. The residues G132, Q133, M184, G330, G349, G358, E425, K427, L432, G452, and Y454 of the binding pocket possessing high betweenness centrality values in apo MetAP-II were thus regarded as potential allosteric sites (Figure 3a).

### Kinetic Analysis

The detailed analysis of the structural dynamics of the apo and ligand-bound MetAP-II was followed by evaluation of the transition states and potential pathways during conformational changes via Markov state model (MSM) analysis. The analysis focused on evaluating changes in the dynamic behavior, role of the active site, and disordered loop region due to ligand binding. MSMs allowed the division of the conformational space into a variety of metastable states with associated structural and kinetic characteristics and also provided a platform for the simulation of the kinetic networks between various metastable states to identify the potential transition pathways.^75,76^ MSMs efficiently sampled transitions between metastable states from several short MD trajectories, whereas MSM networks represented long-time scale dynamics and equilibrium features.^77^ Here, metastable states were generated from MSMs based on the changes observed in the conformational behavior of MetAP-II due to ligand binding to characterize the role of the disordered loop region along with its energetic and kinetic information on transition between states.

The MSM building consisted of several steps involving the selection of different features that can describe the system under study, dimension reduction by either PCA or tICA, and model validation. For adding features, the backbone torsion angles were considered to be the main feature that describe the slow dynamics of the protein,^66^ but in our case, a single feature based on the backbone torsion angles was insufficient to build a converged MSM. Thus, for feature selection, the scoring approach was utilized, which represented a set of scoring functions called the Variational Approach for Markov Process (VAMP-2) that was implemented to determine the best feature mappings and the finest Markovian models for the dynamics from time series data.^67^ In our case, the best feature was chosen from eight different features that were assessed (Figure S1). From these VAMP-2 score plots, we identified the highest VAMP-2 score values for the features based on the distance of the ligand (or bridging hydroxyl group in the case of apo) with all residues of MetAP-II and the distance between the residues of the disordered loop region with the amino acid residues that are within 5 Å of the disordered loop region. Thus, we added all of these features to build MSMs for the apo and ligand-bound systems. Along with these distance features, the backbone phi (φ) and psi (ψ) torsion angles of all residues and the chi 1 angle of the side chain of the active site and the disordered loop region of MetAP-II were also added. The feature-based high dimensionality data were further reduced by PCA into low dimensions for further model generation.

The constructed MSMs of the apo and ligand-bound MetAP-II were further validated based on the Chapman–Kolmogorov (CK) test and the implied time scale (ITS) plot.^78^ Consequently, the ITS plots that validated the generated model for the apo- and the ligand-bound MetAP-II were obtained as a function of lag time (Figures S5 and S8). A lag time of 3 ns was selected for constructing the transition matrix. The five metastable macrostates were retrieved from 50 microstates using the PCCA+ method.^68^ Furthermore, the quantitative properties, including the mean first passage time (MFPT), were computed from the metastable states.

MSM for the apo MetAP-II yielded five metastable states. Each of these states occupies a single energy basin that was used to evaluate the conformational dynamics (Figure S5). The correlated disordered loop region consisting of residue 138–153 in the apo MetAP-II displayed higher mobility, reflecting the significant conformational changes in each metastable state compared to the ligand-bound state (Figure S8), thus corroborating RMSF (Figure S4). Based on the analysis of macrostates, it was revealed that the disordered loop region that is absent in most of the crystal structure^13,39^ behaved like a lid that covered a hidden pocket. The disordered loop was also identified to exist in distinct closed (metastable state 1), semiclosed intermediate state, and open (metastable state 3) conformations (Figure S5a). The distinct closed conformations that populated state 1 matched the crystal structure (Figure S6). The variance between the closed and the open conformations was also confirmed by calculating the Cα-Cα distance between Q141 (residue at the tip of the loop) and E302 of the α4 motif of MetAP-II. The distance between the open state (20.2 Å) exceeds the closed state (10.4 Å) by ∼10 Å (Figure S7). The closed conformation displayed a more compact structure and was involved in the formation of a cryptic pocket near the disordered loop region. In the ligand-bound MetAP-II, the loop was less dynamic and was stabilized in the semiclosed conformation (Figure S9a).

The conformational changes followed a transition path in the identified metastable states of the apo MetAP-II (Figure S 5d). The flux analysis describes pathways of conformational transition from low-populated macrostates to high-populated macrostates based on the forward committer probability on the reversible Markov state process.^79^ Based on the transition path theory, the free energy barrier between macrostate 1 and macrostate 2 was found to be high because it has the longest mean first passage time during the net flux pathways from state 1 to state 2 (Table S3). The flux pathway that visited state 2 showed only a ∼ 7% prevalence at equilibrium. Additionally, the decrease in the free energy going from the source (macrostate 1) to the sink (macrostate 5) visited macrostate 4 that had ∼31% prevalence at equilibrium (Table S4). State 5 was found to be highly populated and had a flexible loop region (Figure S9a). Consequently, state 1 possessed higher loop flexibility than state 5 as deduced from the free energy landscape (FEL).

The transition path theory in ligand-bound MetAP-II also revealed the free energy barriers between each metastable state. The free energy barrier between macrostates 1 and 4 was high due to its longest mean first passage time (Figure S8). The metastable state 4 having the largest mean first passage time in the net flux pathway had only ∼4% prevalence at equilibrium. Moreover, among the five macrostates, the free energy decreased from macrostate 1 to macrostate 5 visiting microstate 2, which showed ∼46% prevalence at the equilibrium, whereas the population density for macrostates 1, 2, and 5 has a prevalence of 15%, 29%, and 20%, respectively (Tables S5 and S6). State 2 has a high density population, and the disordered loop region in this state is more stable than that observed in state 5 (most populated state of apo) (Figures S5a and S8a).

### Interconversion between the Open and Closed Conformations

**Figure 4:**
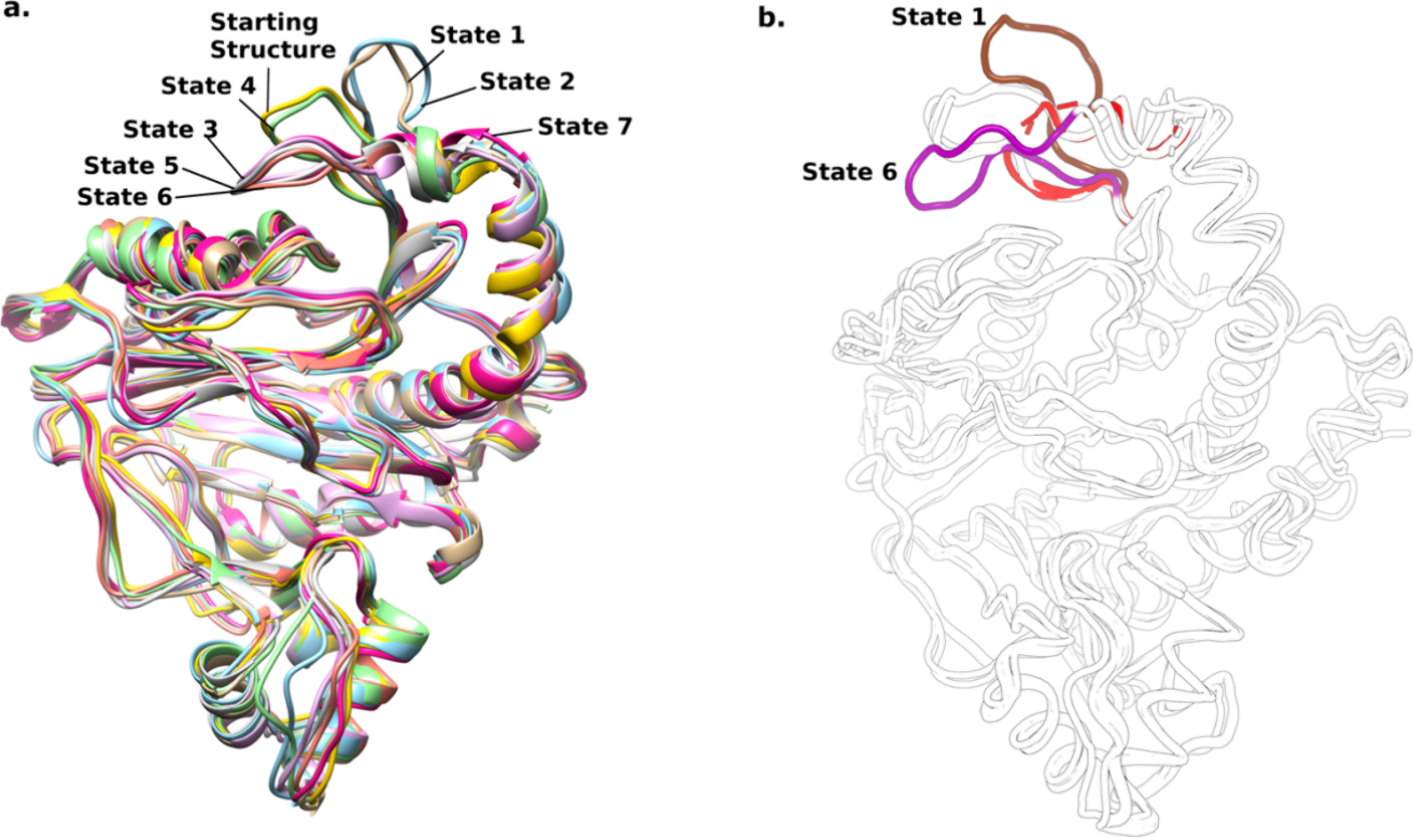
(a) Conformations of the disordered loop in each metastable state obtained from the combined trajectory of apo and ligand-bound MetAP-II, superimposed on the starting crystal structure, representing interconversion of flexible loop region among two states. (b) Superimposed crystal structures of MetAP-II (white) and the two representative conformations (brown and purple) obtained from MSM; dashed red lines represent the missing loop region in X-ray structures (PDB IDs: 6QEJ, 1B59, 1BN5, 1B6A, 1KQ0).

The behavior of the disordered loop region was identified as being more flexible in the apo simulations, where it explored both the open and closed conformations. Thus, to evaluate the interconversion between two states (open and closed conformation of the disordered loop region), the simulation trajectories of the apo and ligand-bound MetAP-II were combined and a new MSM was built. Features were used based on only the backbone phi (φ) and psi (ψ) torsion angles of all residues and the chi 1 angle of the side chain of the active site residues and the disordered loop region. Seven metastable states were then obtained, which presented a clear scenario of the interconversion between the open and closed states, passing through intermediate states. Among seven metastable states, four metastable states (states 3, 5, 6, and 7) displayed closed conformations, whereas two states (states 1 and 2) were in open conformation. State 4 corresponded to a semiclosed intermediate state that perfectly matches the crystal structure with a rmsd of 0.8 Å (Figure 4a).

Moreover, as shown in Table S7, transitions of the conformations from the open to intermediate state occur with average times of 240.04 ns (from state 1) and 288.05 ns (from state 2), indicating that the protein takes a longer time to adopt the intermediate conformation compared to transitioning between open states. Once in the intermediate state (state 4), the protein exhibits relatively shorter transition times to each of the closed conformations (states 3, 5, 6, and 7), with times ranging from 44.53 to 147.01 ns. This suggests that the protein tends to stabilize in one of the closed conformations once it transitions from the intermediate state. Conversely, transitions from closed to intermediate conformations are less frequent, as indicated by the longest MFPT. Furthermore, transitions among closed conformations also show varying transition times, reflecting the dynamics of the system’s conformational landscape.

The states passing from open to intermediate and then close conformation have a prevalence of 7% at the equilibrium, with population densities of 0.5%, 17%, and 9%, respectively). The most populated states were found to be state 5 and state 6 that have population densities of 27% and 21%, respectively. State 1 and state 2 (open conformations) are more energetically favorable than state 4, which displayed the semiclosed conformation and matched the crystal structure (Figure 4b).

To summarize, the analysis of the macrostates obtained from the MSM model identified that the disordered loop region in the apo state of MetAP-II is highly flexible and acts as a lid to cover the cryptic pocket. However, ligand binding at the orthosteric steric reduced the flexibility of this loop region and remained stable in the semiclosed intermediate state (state 2) with a population density of 29%. The volume of the cryptic pocket in open (405 Å^3^), intermediate (279 Å^3^), and close conformations was identified using MDpocket.^70^ The volume from each ensemble was obtained, which confirms the pocket opening and closure interconversion passing through intermediate states (Figure S11).

Subsequently, the betweenness centrality analysis was performed to evaluate the optimal path between Q141 (disordered loop) and E364 (residue participates in the MetAP-II catalytic activity). The results identified that there is a direct route to transfer signal from the identified cryptic pocket to the orthosteric site of MetAP-II (Figure 2c). Only one pathway was identified in the path analysis that takes the direct path Q141 (disordered loop) → T140 → P139 → P138 → Y137 → L464 → Y361 → A362 → E364 (catalytic residue).

**Figure 5:**
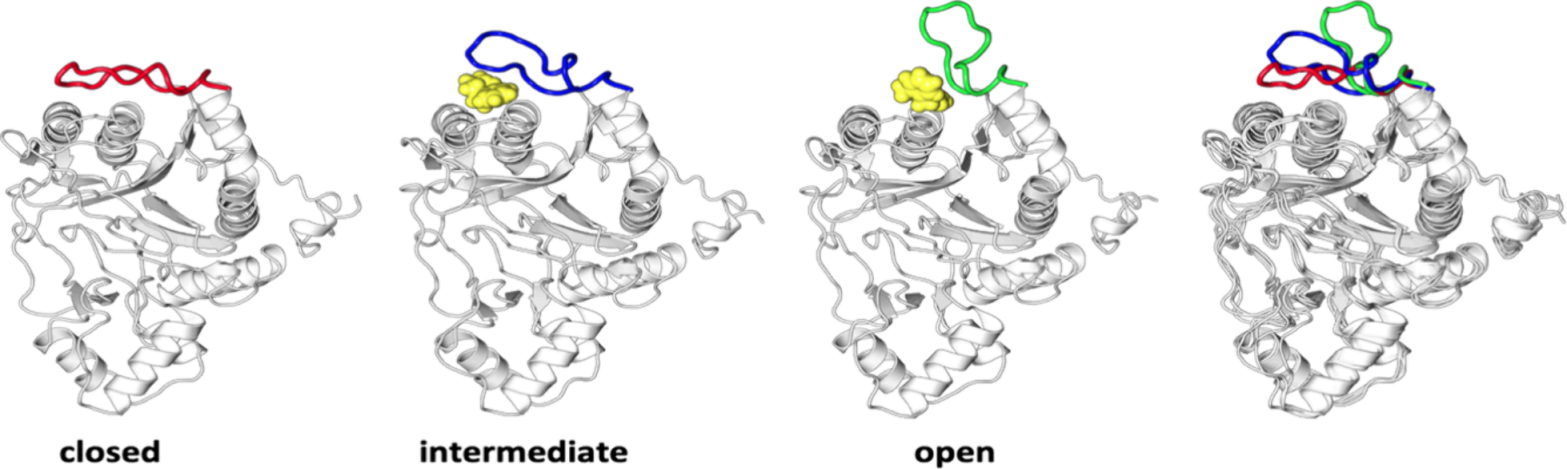
Fragment binding at the disordered loop. Docked fragments (yellow) in the intermediate and the open states at the identified cryptic pocket of MetAP-II. The superimposed conformations of the disordered loop (far right).

Finally, the cryptic pocket was assessed for its druggability using small organic probes. The Ftmap tool uses 16 different sizes of small organic probes to map proteins’ surface, dock, cluster, and rank based on ligand binding energies.^71^ The top-ranked clusters are merged into consensus sites (Css), which represent potential binding sites (Figure 5). The results suggested the pocket to be a hotspot. This was also found to be in good agreement with other analyses, thus confirming the cryptic pocket to be a potential ligand binding site.

Although this proposed allosteric site has been newly identified and has not yet been explored in existing trials, it represents a promising avenue for future drug development efforts for the treatment of cancer. By identifying the cryptic pocket, this research expands the understanding of the MetAP-II structural dynamics, laying the groundwork for potential allosteric modulators that could complement existing orthosteric inhibitors in cancer therapy, including combinatorial therapy (allosteric with an orthosteric drug). Furthermore, the structural insights reported here offer valuable insights that could guide future drug discovery endeavors and enhance therapeutic strategies for cancer treatment involving MetAP-II.

## Conclusions

In this study, we have chosen a potent inhibitor (M8891) of MetAP-II belonging to the class of the new-generation cyclic tartronic diamide scaffold to study the conformational dynamics of MetAP-II after ligand binding. To achieve this, we employed Adaptive Bandit molecular dynamics simulations and built Markov state models to evaluate the structural and dynamical properties of MetAP-II in its apo and ligand-bound (MetAP-II complexed with M8891) states. It is clear from the analysis that the ligand stabilizes MetAP-II. Furthermore, the analysis of metastable states identified that the disordered loop region, which is absent in most of the crystal structures, exists in open, intermediate, and closed conformations in the apo states. The closed conformation of the disordered loop region acts like a lid to a small molecule binding pocket, which also has a high signal communication efficiency. The structural flexibility of this disordered loop is reduced when a ligand is bound in the orthosteric site, 29.7 Å away. This highlights a direct link between the disordered loop and the active site of MetAP-II. The network analysis reveals a single path to transfer signal from the residues in the disordered loop to the active site of MetAP-II. Further analysis based on ligand binding hotspot identification confirms the potential allosteric ligand binding site within this disordered loop region.

## Data Availability

The trajectories, corresponding structure files and the metastable states described in this manuscript can be downloaded from the DOI 10.5281/zenodo.10725653

## Supporting Information Available

Figures S1–S12, including VAMP-2 scores, interactions of M8891 in MetAP-II, RMSD, RMSF, compactness, MSMs of apo, ligand-bound and combined trajectories, comparison with X-ray structures, conformations of the macrostates, pocket analysis and fragment binding; Tables S1–S8 including intramolecular interactions, betweenness centrality, mean first passage times, flux paths, and consensus clusters (PDF)

## References

[ref1] Lowther W. T.; Matthews B. W. Metalloaminopeptidases: Common Functional Themes in Disparate Structural Surroundings. Chem. Rev. 2002, 102, 4581–4608. 10.1021/cr0101757. 12475202

[ref2] Selvakumar P.; Lakshmikuttyamma A.; Dimmock J. R.; Sharma R. K. Methionine Aminopeptidase 2 and Cancer. Biochimica et Biophysica Acta -Reviews on Cancer 2006, 1765, 148–154. 10.1016/j.bbcan.2005.11.001. 16386852

[ref3] Hwang C.-S.; Shemorry A.; Varshavsky A. N-Terminal Acetylation of Cellular Proteins Creates Specific Degradation Signals. Science 2010, 327, 973–977. 10.1126/science.1183147. 20110468 PMC4259118

[ref4] Hu X.; Dang Y.; Tenney K.; Crews P.; Tsai C. W.; Sixt K. M.; Cole P. A.; Liu J. O. Regulation of C-Src Nonreceptor Tyrosine Kinase Activity by Bengamide A through Inhibition of Methionine Aminopeptidases. Chemistry biology 2007, 14, 764–774. 10.1016/j.chembiol.2007.05.010. 17656313 PMC3165037

[ref5] Zhang P.; Nicholson D. E.; Bujnicki J. M.; Su X.; Brendle J. J.; Ferdig M.; Kyle D. E.; Milhous W. K.; Chiang P. K. Angiogenesis Inhibitors Specific for Methionine Aminopeptidase 2 as Drugs for Malaria and Leishmaniasis. Journal of biomedical science 2002, 9, 34–40. 10.1007/BF02256576. 11810023

[ref6] Arfin S. M.; Kendall R. L.; Hall L.; Weaver L. H.; Stewart A. E.; Matthews B. W.; Bradshaw R. A. Eukaryotic Methionyl Aminopeptidases: Two Classes of Cobalt-Dependent Enzymes. Proc. Natl. Acad. Sci. U. S. A. 1995, 92, 7714–7718. 10.1073/pnas.92.17.7714. 7644482 PMC41216

[ref7] Yeh J.-R. J. A Study of the Anti-Angiogenic Agent TNP-470: Revealing a Unique Mechanism of Endothelial Cell Cycle Control and Genetic Targeting of Methionine Aminopeptidase-2, the Binding Protein of TNP-470; Yale University 2001.

[ref8] Hu X.; Addlagatta A.; Lu J.; Matthews B. W.; Liu J. O. Elucidation of the Function of Type 1 Human Methionine Aminopeptidase during Cell Cycle Progression. Proc. Natl. Acad. Sci. U. S. A. 2006, 103, 18148–18153. 10.1073/pnas.0608389103. 17114291 PMC1838721

[ref9] Zhang Y.; Griffith E. C.; Sage J.; Jacks T.; Liu J. O. Cell Cycle Inhibition by the Anti-Angiogenic Agent TNP-470 Is Mediated by P53 and p21WAF1/CIP1. Proc. Natl. Acad. Sci. U. S. A. 2000, 97, 6427–6432. 10.1073/pnas.97.12.6427. 10841547 PMC18619

[ref10] Yang G.; Kirkpatrick R. B.; Ho T.; Zhang G. F.; Liang P. H.; Johanson K. O.; Casper D. J.; Doyle M. L.; Marinox J. P.; Thompson S. K.; Chen W.; Tew D. G.; Meek T. D. Steady-State Kinetic Characterization of Substrates and Metal-Ion Specificities of the Full-Length and N-Terminally Truncated Recombinant Human Methionine Aminopeptidases (Type 2). Biochemistry 2001, 40, 10645–10654. 10.1021/bi010806r. 11524009

[ref11] Kishor C.; Arya T.; Reddi R.; Chen X.; Saddanapu V.; Marapaka A. K.; Gumpena R.; Ma D.; Liu J. O.; Addlagatta A. Identification, Biochemical and Structural Evaluation of Species-Specific Inhibitors against Type I Methionine Aminopeptidases. Journal of medicinal chemistry 2013, 56, 5295–5305. 10.1021/jm400395p. 23767698

[ref12] Addlagatta A.; Hu X.; Liu J. O.; Matthews B. W. Structural Basis for the Functional Differences between Type I and Type II Human Methionine Aminopeptidases. Biochemistry 2005, 44, 14741–14749. 10.1021/bi051691k. 16274222

[ref13] Liu S.; Widom J.; Kemp C. W.; Crews C. M.; Clardy J. Structure of Human Methionine Aminopeptidase-2 Complexed with Fumagillin. Science 1998, 282, 1324–1327. 10.1126/science.282.5392.1324. 9812898

[ref14] Datta B. Roles of P67/MetAP2 as a Tumor Suppressor. Biochim. Biophys. Acta BBA - Rev. Cancer 2009, 1796 (2), 281–292. 10.1016/j.bbcan.2009.08.002. 19716858

[ref15] Bradshaw R. A.; Brickey W. W.; Walker K. W. N-Terminal Processing: The Methionine Aminopeptidase and Nα-Acetyl Transferase Families. Trends in biochemical sciences 1998, 23, 263–267. 10.1016/S0968-0004(98)01227-4. 9697417

[ref16] Lowther W. T.; Orville A. M.; Madden D. T.; Lim S.; Rich D. H.; Matthews B. W. Escherichia Coli Methionine Aminopeptidase: Implications of Crystallographic Analyses of the Native, Mutant, and Inhibited Enzymes for the Mechanism of Catalysis. Biochemistry 1999, 38, 7678–7688. 10.1021/bi990684r. 10387007

[ref17] Evdokimov A. G.; Pokross M.; Walter R. L.; Mekel M.; Barnett B. L.; Amburgey J.; Seibel W. L.; Soper S. J.; Djung J. F.; Fairweather N.; Diven C.; Rastogi V.; Grinius L.; Klanke C.; Siehnel R.; Twinem T.; Andrews R.; Curnow A. Serendipitous Discovery of Novel Bacterial Methionine Aminopeptidase Inhibitors. Proteins: Struct., Funct., Bioinf. 2007, 66, 538–546. 10.1002/prot.21207. 17120228

[ref18] Brown D. A.; Errington W.; Glass W.; Haase W.; Kemp T.; Nimir H.; Ostrovsky S.; Werner R. Magnetic, Spectroscopic, and Structural Studies of Dicobalt Hydroxamates and Model Hydrolases. Inorg. Chem. 2001, 40, 5962–5971. 10.1021/ic0103345. 11681912

[ref19] Sherman F.; Stewart J. W.; Tsunasawa S. Methionine or Not Methionine at the Beginning of a Protein. Bioessays 1985, 3, 27–31. 10.1002/bies.950030108. 3024631

[ref20] Lowther W. T.; Matthews B. W. Structure and Function of the Methionine Aminopeptidases. Biochimica et Biophysica Acta -Protein Structure, Molecular Enzymology 2000, 1477, 157–167. 10.1016/S0167-4838(99)00271-X. 10708856

[ref21] Ben-Bassat A.; Bauer K.; Chang S.-Y.; Myambo K.; Boosman A.; Chang S. Processing of the Initiation Methionine from Proteins: Properties of the Escherichia Coli Methionine Aminopeptidase and Its Gene Structure. Journal of bacteriology 1987, 169, 751–757. 10.1128/jb.169.2.751-757.1987. 3027045 PMC211843

[ref22] Chang Y.; Teichert U.; Smith J. Molecular Cloning, Sequencing, Deletion, and Overexpression of a Methionine Aminopeptidase Gene from Saccharomyces Cerevisiae. J. Biol. Chem. 1992, 267, 8007–8011. 10.1016/S0021-9258(18)42400-3. 1569059

[ref23] Li X.; Chang Y.-H. Evidence That the Human Homologue of a Rat Initiation Factor-2 Associated Protein (P67) Is a Methionine Aminopeptidase. Biochemical biophysical research communications 1996, 227, 152–159. 10.1006/bbrc.1996.1482. 8858118

[ref24] Wang J.; Sheppard G. S.; Lou P.; Kawai M.; Park C.; Egan D. A.; Schneider A.; Bouska J.; Lesniewski R.; Henkin J. Physiologically Relevant Metal Cofactor for Methionine Aminopeptidase-2 Is Manganese. Biochemistry 2003, 42, 5035–5042. 10.1021/bi020670c. 12718546

[ref25] Konisti S.; Kiriakidis S.; Paleolog E. M. J A.; Cellular V.; Health M. M. Angiogenesis in Rheumatoid Arthritis. Diseases 2013, 339–365. 10.1186/ar575.

[ref26] Kanno T.; Endo H.; Takeuchi K.; Morishita Y.; Fukayama M.; Mori S. High Expression of Methionine Aminopeptidase Type 2 in Germinal Center B Cells and Their Neoplastic Counterparts. Laboratory investigation 2002, 82, 893–901. 10.1097/01.LAB.0000020419.25365.C4. 12118091

[ref27] Selvakumar P.; Lakshmikuttyamma A.; Shrivastav A.; Das S. B.; Dimmock J. R.; Sharma R. K. Potential Role of N-Myristoyltransferase in Cancer. Progress in lipid research 2007, 46, 1–36. 10.1016/j.plipres.2006.05.002. 16846646

[ref28] Griffith E. C.; Su Z.; Turk B. E.; Chen S.; Chang Y.-H.; Wu Z.; Biemann K.; Liu J. O. Methionine Aminopeptidase (Type 2) Is the Common Target for Angiogenesis Inhibitors AGM-1470 and Ovalicin. Chemistry biology 1997, 4, 461–471. 10.1016/S1074-5521(97)90198-8. 9224570

[ref29] Shusterman S.; Maris J. M. Prospects for Therapeutic Inhibition of Neuroblastoma Angiogenesis. Cancer letters 2005, 228, 171–179. 10.1016/j.canlet.2005.01.049. 15927358

[ref30] Griffith E. C.; Su Z.; Niwayama S.; Ramsay C. A.; Chang Y.-H.; Liu J. O. Molecular Recognition of Angiogenesis Inhibitors Fumagillin and Ovalicin by Methionine Aminopeptidase 2. Proc. Natl. Acad. Sci. U. S. A. 1998, 95, 15183–15188. 10.1073/pnas.95.26.15183. 9860943 PMC28017

[ref31] Yin S.-Q.; Wang J.-J.; Zhang C.-M.; Liu Z.-P. The Development of MetAP-2 Inhibitors in Cancer Treatment. Curr. Med. Chem. 2012, 19, 1021–1035. 10.2174/092986712799320709. 22229417

[ref32] Bernier S. G.; Lazarus D. D.; Clark E.; Doyle B.; Labenski M. T.; Thompson C. D.; Westlin W. F.; Hannig G. A Methionine Aminopeptidase-2 Inhibitor, PPI-2458, for the Treatment of Rheumatoid Arthritis. Proc. Natl. Acad. Sci. U. S. A. 2004, 101, 10768–10773. 10.1073/pnas.0404105101. 15249666 PMC490009

[ref33] Kusaka M.; Sudo K.; Fujita T.; Marui S.; Itoh F.; Ingber D.; Folkman J. Potent Anti-Angiogenic Action of AGM-1470: Comparison to the Fumagillin Parent. Biochemical biophysical research communications 1991, 174, 1070–1076. 10.1016/0006-291X(91)91529-L. 1705118

[ref34] Bernier S. G.; Westlin W. F.; Hannig G. Fumagillin Class Inhibitors of Methionine Aminopeptidase-2. Drugs Future 2005, 30, 0497–508. 10.1358/dof.2005.030.05.895807.

[ref35] Towbin H.; Bair K. W.; DeCaprio J. A.; Eck M. J.; Kim S.; Kinder F. R.; Morollo A.; Mueller D. R.; Schindler P.; Song H. K.; van Oostrum J.; Versace R. W.; Voshol H.; Wood J.; Zabludoff S.; Phillips P. E. Proteomics-Based Target Identification: Bengamides as a New Class of Methionine Aminopeptidase Inhibitors. J. Biol. Chem. 2003, 278, 52964–52971. 10.1074/jbc.M309039200. 14534293

[ref36] Kawai M.; BaMaung N. Y.; Fidanze S. D.; Erickson S. A.; Tedrow J. S.; Sanders W. J.; Vasudevan A.; Park C.; Hutchins C.; Comess K. M.; Kalvin D.; Wang J.; Zhang Q.; Lou P.; Tucker-Garcia L.; Bouska J.; Bell R. L.; Lesniewski R.; Henkin J.; Sheppard G. S. Development of Sulfonamide Compounds as Potent Methionine Aminopeptidase Type II Inhibitors with Antiproliferative Properties. Bioorg. Med. Chem. Lett. 2006, 16, 3574–3577. 10.1016/j.bmcl.2006.03.085. 16632353

[ref37] Sheppard G. S.; Wang J.; Kawai M.; BaMaung N. Y.; Craig R. A.; Erickson S. A.; Lynch L.; Patel J.; Yang F.; Searle X. B.; Lou P.; Park C.; Kim K. H.; Henkin J.; Lesniewski R. 3-Amino-2-Hydroxyamides and Related Compounds as Inhibitors of Methionine Aminopeptidase-2. Bioorg. Med. Chem. Lett. 2004, 14, 865–868. 10.1016/j.bmcl.2003.12.031. 15012983

[ref38] Marino J. P.; Fisher P. W.; Hofmann G. A.; Kirkpatrick R. B.; Janson C. A.; Johnson R. K.; Ma C.; Mattern M.; Meek T. D.; Ryan M. D.; Schulz C.; Smith W. W.; Tew D. G.; Tomazek T. A.; Veber D. F.; Xiong W. C.; Yamamoto Y.; Yamashita K.; Yang G.; Thompson S. K. Highly Potent Inhibitors of Methionine Aminopeptidase-2 Based on a 1, 2, 4-Triazole Pharmacophore. J. Med. Chem. 2007, 50, 3777–3785. 10.1021/jm061182w. 17636946

[ref39] Heinrich T.; Seenisamy J.; Blume B.; Bomke J.; Calderini M.; Eckert U.; Friese-Hamim M.; Kohl R.; Lehmann M.; Leuthner B.; Musil D.; Rohdich F.; Zenke F. T. Discovery and Structure-Based Optimization of next-Generation Reversible Methionine Aminopeptidase-2 (MetAP-2) Inhibitors. J. Med. Chem. 2019, 62, 5025–5039. 10.1021/acs.jmedchem.9b00041. 30939017

[ref40] Pérez A.; Herrera-Nieto P.; Doerr S.; De Fabritiis G. AdaptiveBandit: A Multi-Armed Bandit Framework for Adaptive Sampling in Molecular Simulations. J. Chem. Theory Comput. 2020, 16, 4685–4693. 10.1021/acs.jctc.0c00205. 32539384

[ref41] Sheppard G. S.; Wang J.; Kawai M.; Fidanze S. D.; BaMaung N. Y.; Erickson S. A.; Barnes D. M.; Tedrow J. S.; Kolaczkowski L.; Vasudevan A.; Park D. C.; Wang G. T.; Sanders W. J.; Mantei R. A.; Palazzo F.; Tucker-Garcia L.; Lou P.; Zhang Q.; Park C. H.; Kim K. H.; Petros A.; Olejniczak E.; Nettesheim D.; Hajduk P.; Henkin J.; Lesniewski R.; Davidsen S. K.; Bell R. L. Discovery and Optimization of Anthranilic Acid Sulfonamides as Inhibitors of Methionine Aminopeptidase-2: A Structural Basis for the Reduction of Albumin Binding. J. Med. Chem. 2006, 49, 3832–3849. 10.1021/jm0601001. 16789740

[ref42] Klein C. D.; Schiffmann R.; Folkers G.; Piana S.; Röthlisberger U. Protonation States of Methionine Aminopeptidase and Their Relevance for Inhibitor Binding and Catalytic Activity. J. Biol. Chem. 2003, 278, 47862–47867. 10.1074/jbc.M305325200. 14514693

[ref43] Jørgensen A. T.; Norrby P. O.; Liljefors T. Investigation of the Metal Binding Site in Methionine Aminopeptidase by Density Functional Theory. J. Comput.-Aided Mol. Des. 2002, 16, 167–179. 10.1023/A:1020119527789. 12363216

[ref44] Babu C. S.; Lim C. Empirical Force Fields for Biologically Active Divalent Metal Cations in Water. J. Phys. Chem. A 2006, 110, 691–699. 10.1021/jp054177x. 16405342

[ref45] Frisch M.; Trucks G.; Schlegel H.; Scuseria G.; Robb M.; Cheeseman J.; Scalmani G.; Barone V.; Mennucci B.; Petersson G. Uranyl Extraction by N, N-Dialkylamide Ligands Studied by Static and Dynamic DFT Simulations. Gaussian 09; Gaussian Inc., 2009.

[ref46] Hertwig R. H.; Koch W. On the Parameterization of the Local Correlation Functional. What Is Becke-3-LYP?. Chem. Phys. Lett. 1997, 268, 345–351. 10.1016/S0009-2614(97)00207-8.

[ref47] Bayly C. I.; Cieplak P.; Cornell W.; Kollman P. A. A Well-Behaved Electrostatic Potential Based Method Using Charge Restraints for Deriving Atomic Charges: The RESP Model. J. Phys. Chem. 1993, 97, 10269–10280. 10.1021/j100142a004.

[ref48] Wang J.; Wolf R. M.; Caldwell J. W.; Kollman P. A.; Case D. A. Development and Testing of a General Amber Force Field. J. Comput. Chem. 2004, 25, 1157–1174. 10.1002/jcc.20035. 15116359

[ref49] Duarte F.; Bauer P.; Barrozo A.; Amrein B. A.; Purg M.; Åqvist J.; Kamerlin S. C. L. Force Field Independent Metal Parameters Using a Nonbonded Dummy Model. J. Phys. Chem. B 2014, 118, 4351–4362. 10.1021/jp501737x. 24670003 PMC4180081

[ref50] Doerr S.; Harvey M.; Noé F.; De Fabritiis G. HTMD: High-Throughput Molecular Dynamics for Molecular Discovery. J. Chem. Theory Comput. 2016, 12, 1845–1852. 10.1021/acs.jctc.6b00049. 26949976

[ref51] Case D. A.; Aktulga H. M.; Belfon K.; Cerutti D. S.; Cisneros G. A.; Cruzeiro V. W. D.; Forouzesh N.; Giese T. J.; Gotz A. W.; Gohlke H.; Izadi S.; Kasavajhala K.; Kaymak M. C.; King E.; Kurtzman T.; Lee T. S.; Li P.; Liu J.; Luchko T.; Luo R.; Manathunga M.; Machado M. R.; Nguyen H. M.; O’Hearn K. A.; Onufriev A. V.; Pan F.; Pantano S.; Qi R.; Rahnamoun A.; Risheh A.; Schott-Verdugo S.; Shajan A.; Swails J.; Wang J.; Wei H.; Wu X.; Wu Y.; Zhang S.; Zhao S.; Zhu Q.; Cheatham T. E. 3rd; Roe D. R.; Roitberg A.; Simmerling C.; York D. M.; Nagan M. C.; Merz K. M. Jr. AmberTools. J. Chem. Inf Model 2023, 63 (20), 6183–6191. 10.1021/acs.jcim.3c01153. 37805934 PMC10598796

[ref52] Jorgensen W. L.; Chandrasekhar J.; Madura J. D.; Impey R. W.; Klein M. L. Comparison of Simple Potential Functions for Simulating Liquid Water. J. Chem. Phys. 1983, 79, 926–935. 10.1063/1.445869.

[ref53] Mark P.; Nilsson L. Structure and Dynamics of the TIP3P, SPC, and SPC/E Water Models at 298 K. J. Phys. Chem. A 2001, 105, 9954–9960. 10.1021/jp003020w.

[ref54] Harvey M. J.; Giupponi G.; Fabritiis G. D. ACEMD: Accelerating Biomolecular Dynamics in the Microsecond Time Scale. J. Chem. Theory Comput. 2009, 5, 1632–1639. 10.1021/ct9000685. 26609855

[ref55] Roe D. R.; Cheatham T. E. III PTRAJ and CPPTRAJ: Software for Processing and Analysis of Molecular Dynamics Trajectory Data. J. Chem. Theory Comput. 2013, 9, 3084–3095. 10.1021/ct400341p. 26583988

[ref56] Michaud-Agrawal N.; Denning E. J.; Woolf T. B.; Beckstein O. MDAnalysis: A Toolkit for the Analysis of Molecular Dynamics Simulations. Journal of computational chemistry 2011, 32, 2319–2327. 10.1002/jcc.21787. 21500218 PMC3144279

[ref57] Humphrey W.; Dalke A.; Schulten K. VMD: Visual Molecular Dynamics. J. Mol. Graphics 1996, 14, 33–38. 10.1016/0263-7855(96)00018-5. 8744570

[ref58] Pettersen E. F.; Goddard T. D.; Huang C. C.; Couch G. S.; Greenblatt D. M.; Meng E. C.; Ferrin T. E. UCSF Chimera—a Visualization System for Exploratory Research and Analysis. Journal of computational chemistry 2004, 25, 1605–1612. 10.1002/jcc.20084. 15264254

[ref59] Tomasello G.; Armenia I.; Molla G. The Protein Imager: A Full-Featured Online Molecular Viewer Interface with Server-Side HQ-Rendering Capabilities. Bioinformatics 2020, 36 (9), 2909–2911. 10.1093/bioinformatics/btaa009. 31930403

[ref60] Glasser L.; Herráez A.; Hanson R. M. Interactive 3D Phase Diagrams Using Jmol. J. Chem. Educ. 2009, 86, 566 10.1021/ed086p566.

[ref61] Mercadante D.; Gräter F.; Daday C. CONAN: A Tool to Decode Dynamical Information from Molecular Interaction Maps. Biophysical journal 2018, 114, 1267–1273. 10.1016/j.bpj.2018.01.033. 29590584 PMC5883949

[ref62] Brown D. K.; Penkler D. L.; Sheik Amamuddy O.; Ross C.; Atilgan A. R.; Atilgan C.; Tastan Bishop Ö. MD-TASK: A Software Suite for Analyzing Molecular Dynamics Trajectories. Bioinformatics 2017, 33, 2768–2771. 10.1093/bioinformatics/btx349. 28575169 PMC5860072

[ref63] del Sol A.; O'Meara P. Small-world Network Approach to Identify Key Residues in Protein–Protein Interaction. Proteins: Struct., Funct., Bioinf. 2005, 58, 672–682. 10.1002/prot.20348. 15617065

[ref64] Li H.; Chang Y.-Y.; Lee J. Y.; Bahar I.; Yang L.-W. DynOmics: Dynamics of Structural Proteome and Beyond. Nucleic acids research 2017, 45, W374–W380. 10.1093/nar/gkx385. 28472330 PMC5793847

[ref65] Scherer M. K.; Trendelkamp-Schroer B.; Paul F.; Pérez-Hernández G.; Hoffmann M.; Plattner N.; Wehmeyer C.; Prinz J.-H.; Noé F. PyEMMA 2: A Software Package for Estimation, Validation, and Analysis of Markov Models. J. Chem. Theory Comput. 2015, 11, 5525–5542. 10.1021/acs.jctc.5b00743. 26574340

[ref66] Bastolla U.; Dehouck Y. Can Conformational Changes of Proteins Be Represented in Torsion Angle Space? A Study with Rescaled Ridge Regression. J. Chem. Inf. Model. 2019, 59, 4929–4941. 10.1021/acs.jcim.9b00627. 31600071

[ref67] Wu H.; Noé F. Variational Approach for Learning Markov Processes from Time Series Data. J. Nonlinear Sci. 2020, 30, 23–66. 10.1007/s00332-019-09567-y.

[ref68] Deuflhard P.; Weber M. Robust Perron Cluster Analysis in Conformation Dynamics. Linear algebra and its applications 2005, 398, 161–184. 10.1016/j.laa.2004.10.026.

[ref69] Held M.; Metzner P.; Prinz J.-H.; Noé F. Mechanisms of Protein-Ligand Association and Its Modulation by Protein Mutations. Biophysical journal 2011, 100, 701–710. 10.1016/j.bpj.2010.12.3699. 21281585 PMC3030248

[ref70] Schmidtke P.; Bidon-Chanal A.; Luque F. J.; Barril X. MDpocket: Open-Source Cavity Detection and Characterization on Molecular Dynamics Trajectories. Bioinformatics 2011, 27, 3276–3285. 10.1093/bioinformatics/btr550. 21967761

[ref71] Kozakov D.; Grove L. E.; Hall D. R.; Bohnuud T.; Mottarella S. E.; Luo L.; Xia B.; Beglov D.; Vajda S. The FTMap Family of Web Servers for Determining and Characterizing Ligand-Binding Hot Spots of Proteins. Nature protocols 2015, 10, 733–755. 10.1038/nprot.2015.043. 25855957 PMC4762777

[ref72] Van Wart A. T.; Durrant J.; Votapka L.; Amaro R. E. Weighted Implementation of Suboptimal Paths (WISP): An Optimized Algorithm and Tool for Dynamical Network Analysis. J. Chem. Theory Comput. 2014, 10, 511–517. 10.1021/ct4008603. 24803851 PMC3958135

[ref73] Carducci M. A.; Wang D.; Habermehl C.; Bŏdding M.; Rohdich F.; Lignet F.; Duecker K.; Karpenko O.; Pudelko L.; Gimmi C.; LoRusso P. A First-in-Human, Dose-Escalation Study of the Methionine Aminopeptidase 2 Inhibitor M8891 in Patients with Advanced Solid Tumors. Cancer Res. Commun. 2023, 3, 1638–1647. 10.1158/2767-9764.CRC-23-0048. 37637935 PMC10448909

[ref74] Pu L.; Govindaraj R. G.; Lemoine J. M.; Wu H.-C.; Brylinski M. DeepDrug3D: Classification of Ligand-Binding Pockets in Proteins with a Convolutional Neural Network. PLoS computational biology 2019, 15, e1006718 10.1371/journal.pcbi.1006718. 30716081 PMC6375647

[ref75] Chodera J. D.; Noé F. Markov State Models of Biomolecular Conformational Dynamics. Curr. Opin. Struct. Biol. 2014, 25, 135–144. 10.1016/j.sbi.2014.04.002. 24836551 PMC4124001

[ref76] Trendelkamp-Schroer B.; Wu H.; Paul F.; Noé F. Estimation and Uncertainty of Reversible Markov Models. J. Chem. Phys., 2015, 143. 10.1063/1.4934536 26547152

[ref77] Shukla D.; Hernández C. X.; Weber J. K.; Pande V. S. Markov State Models Provide Insights into Dynamic Modulation of Protein Function. Accounts of chemical research 2015, 48, 414–422. 10.1021/ar5002999. 25625937 PMC4333613

[ref78] Prinz J.-H.; Wu H.; Sarich M.; Keller B.; Senne M.; Held M.; Chodera J. D.; Schütte C.; Noé F. Markov Models of Molecular Kinetics: Generation and Validation. J. Chem. Phys., 2011, 134. 10.1063/1.3565032 21548671

[ref79] Meng Y.; Shukla D.; Pande V. S.; Roux B. Transition Path Theory Analysis of C-Src Kinase Activation. Proc. Natl. Acad. Sci. U. S. A. 2016, 113, 9193–9198. 10.1073/pnas.1602790113. 27482115 PMC4995974

