# Supplementary material for: Identification of a Cryptic Pocket in Methionine Aminopeptidase-II Using Adaptive Bandit Molecular Dynamics Simulations and Markov State Models
Source: ACS Omega. 2024 Jun 18;9(26):28534–45. doi: 10.1021/acsomega.4c02516 (PMC11223136; doi:10.1021/acsomega.4c02516)
Supplement: Supplementary file 1 — ao4c02516_si_001.pdf [file ao4c02516_si_001.pdf]

Identification of a Cryptic Pocket in Methionine Aminopeptidase-II using Adaptive Bandit Molecular Dynamics Simulations and Markov State Models

SUPPLEMENTARY INFORMATION

Rubina[a], Syed Tarique Moin[a], Shozeb Haider[b,c,*]

[a] Third World Center for Science and Technology, H.E.J. Research Institute of Chemistry, International Center for Chemical and Biological Sciences, University of Karachi, Karachi-75270, Pakistan

[b] UCL School of Pharmacy, University College London, London, WC1N 1AX, U.K.;
[c] UCL Centre for Advanced Research Computing, University College London, London, WC1H 9RN, U.K.

Corresponding Author

https://orcid.org/0000-0003-2650-2925;
Email: shozeb.haider@ucl.ac.uk

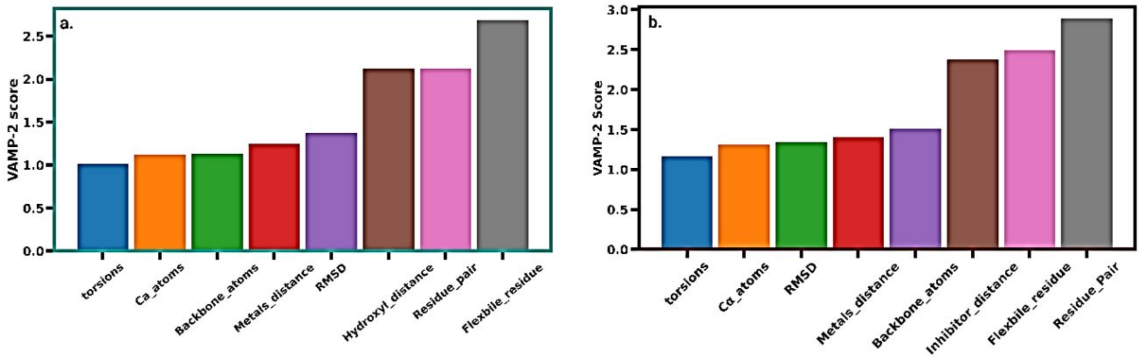

Figure S1: VAMP-2 score for different features assesed in MetAP-II. Torsion (phi and psi), Cα-atoms (position of Cα-atoms), Backbone atoms (position of backbone atoms), Metal distance (distance of residues from the metal within 5Å), RMSD (root mean square deviation), Hydroxyl/inhibitor distance (distance of residues from hydroxyl/ligand within 5 Å), Residue pair (distance of hydroxyl or ligand with each residues), and flexible residues (disordered loop region). These features were tested for (a) apo and (b) ligand-bound MetAP-II.

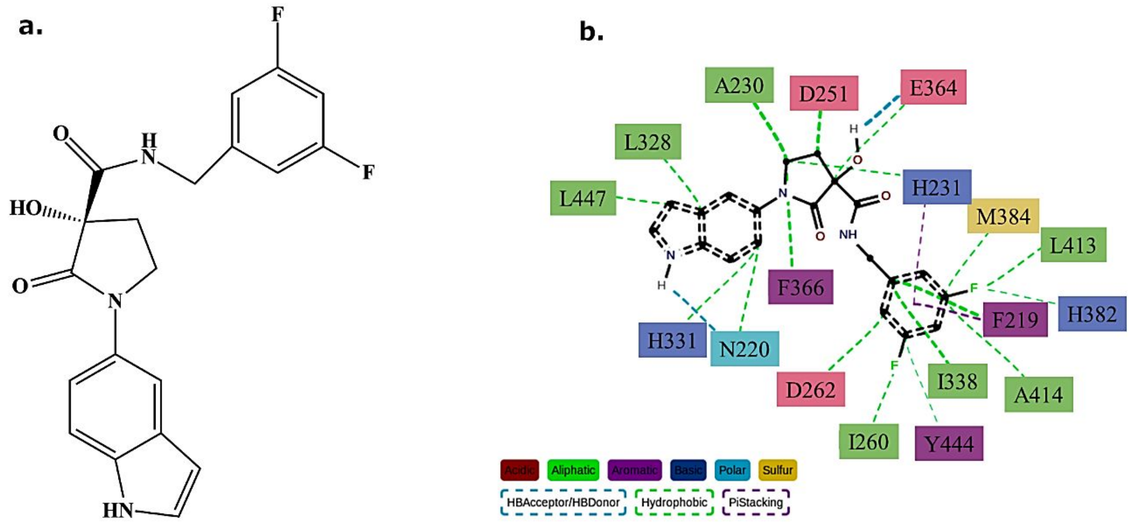

Figure S2: Interaction of M8891 in MetAP-II. (a) Chemical structure of M8891, (b) interactions made by M8891. E364 acts as a hydrogen bond donor, other aromatic residues are involved in the π-π stacking, and hydrogen bonding.

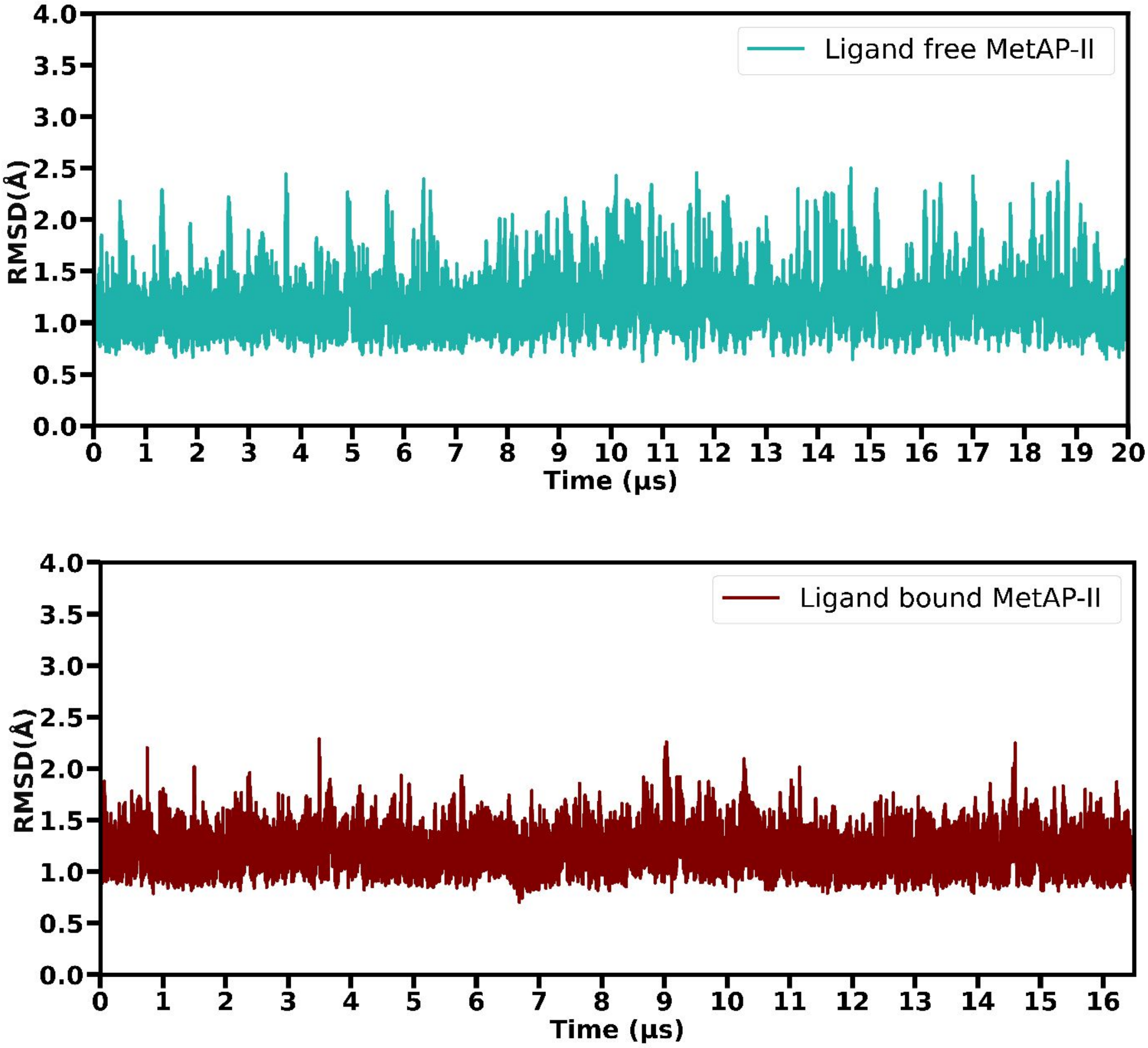

Figure S3: Root Mean Square Deviation (RMSD) of Cα backbone RMSD of apo MetAP-II, and ligand-bound MetAP-II.

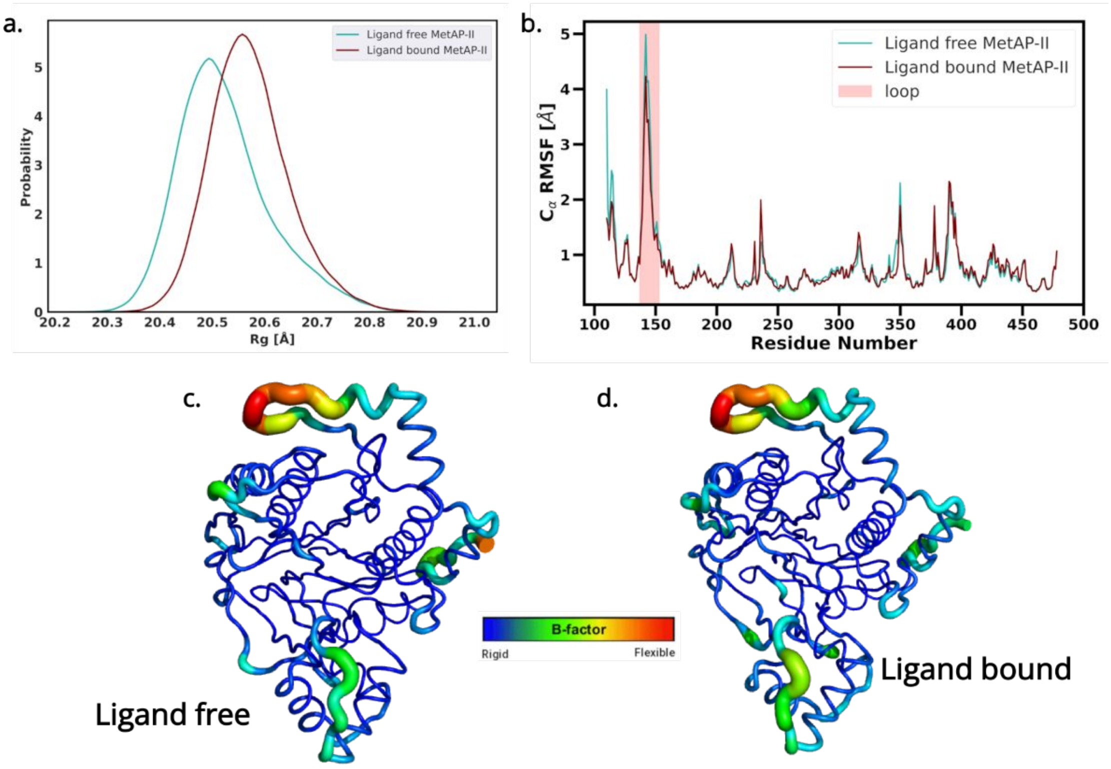

Figure S4: Compactness of MetAP-II in Adaptive sampling simulations (a) probability plot of the radius of gyration (Rg) of both systems, suggested compactness of the ligand-bound MetAP-II, (b) Cα Root Mean Square Fluctuation (RMSF) of each residue in apo and ligand-bound MetAP-II. (c) RMSF representation on the strucure of apo, and (d) ligand-bound MetAP-II. The thick red ribbon represents a high RMSF value, whereas thin blue indicate stable regions. The terminal and disordered loop regions display high RMSF values, highlighting the dynamic nature of the sturcture.

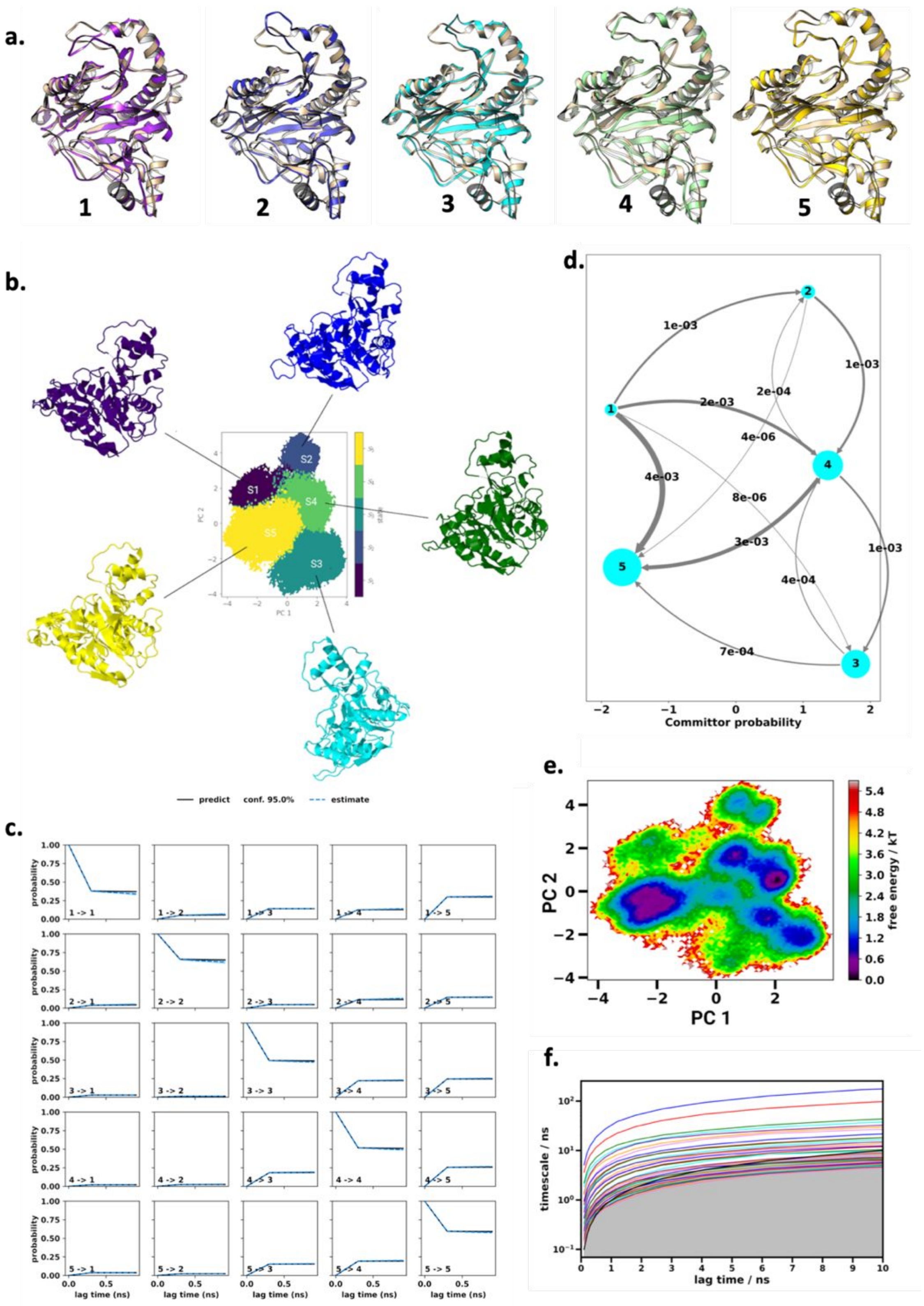

Figure S5: Markov State Model (MSM) of apo MetAP-II. (a) each metastable state superimposed on the crystal structure (tan); (b) macrostate distribution projected on the first two principal components along with their representatives; (c) CK test plot; (d) Transition path theory analysis; net flux plot shows the probabilities of each transition in the relevant direction per unit time;(e) Free Energy Landscape; (f) Implied timescale plot.

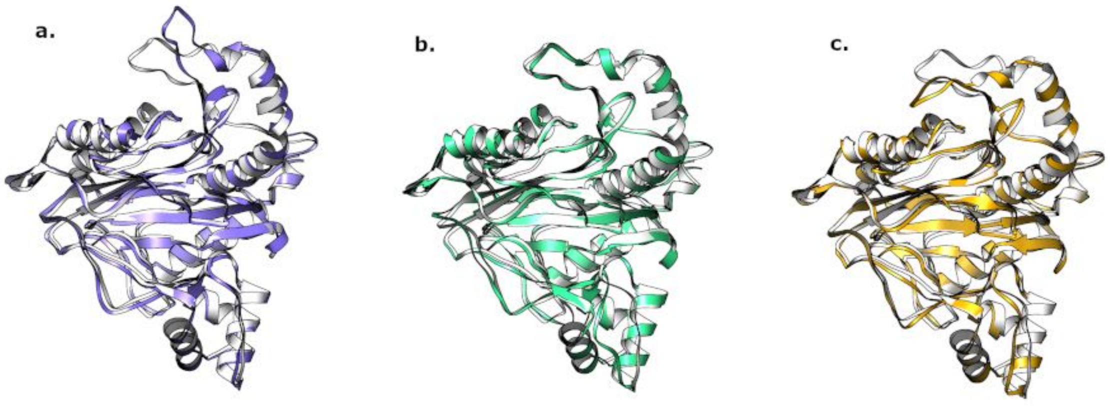

Figure S6: The X-ray structure of apo MetAP-II (PDB ID: 1yw9 -White) superimposed with the extracted (a) open, (b) intermediate and (c) closed conformations of the disordered loop region.

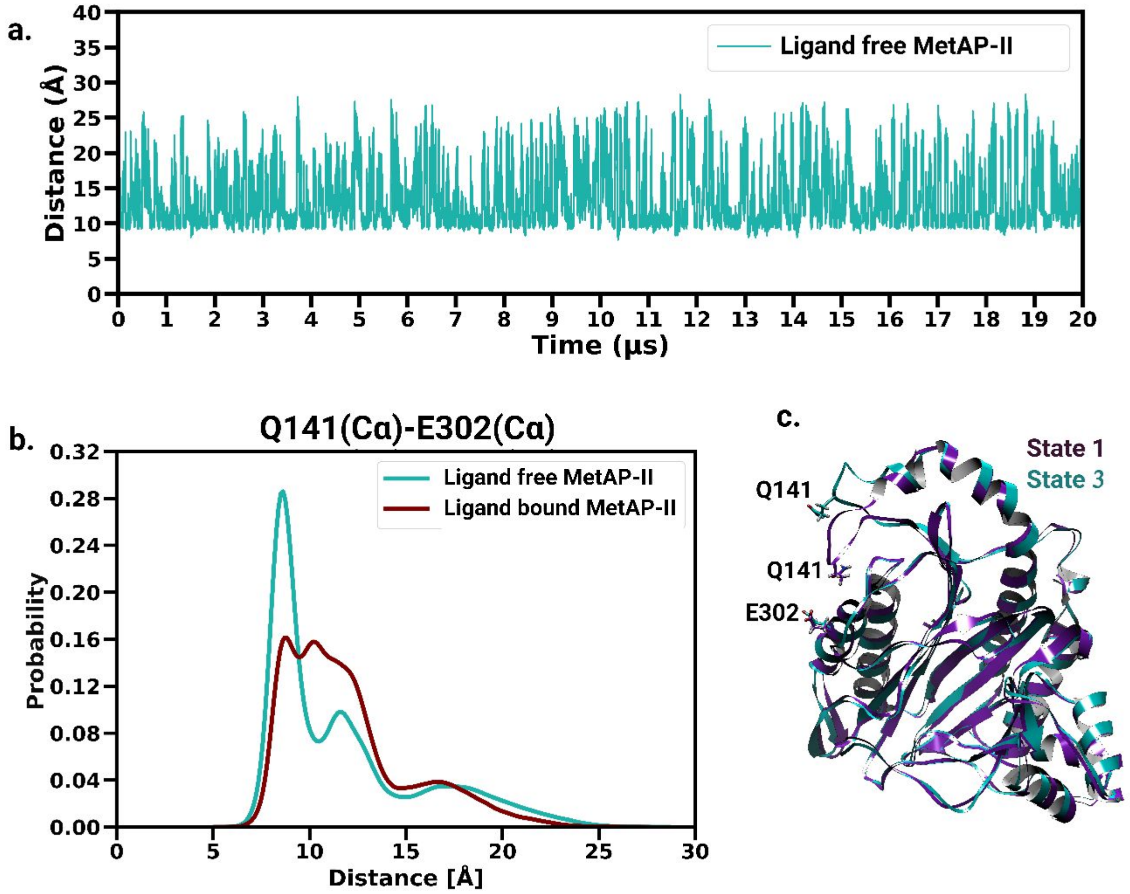

Figure S7: Conformation in the apo macrostates (a) Distance between Q141-E302 in apo state throughout the simulation time; (b) Probability plot of distance in apo and ligand-bound states of MetAP-II and (c) representative structures of macrostate 1 (closed) and macrostate 3 (open) in apo MetAP-II.

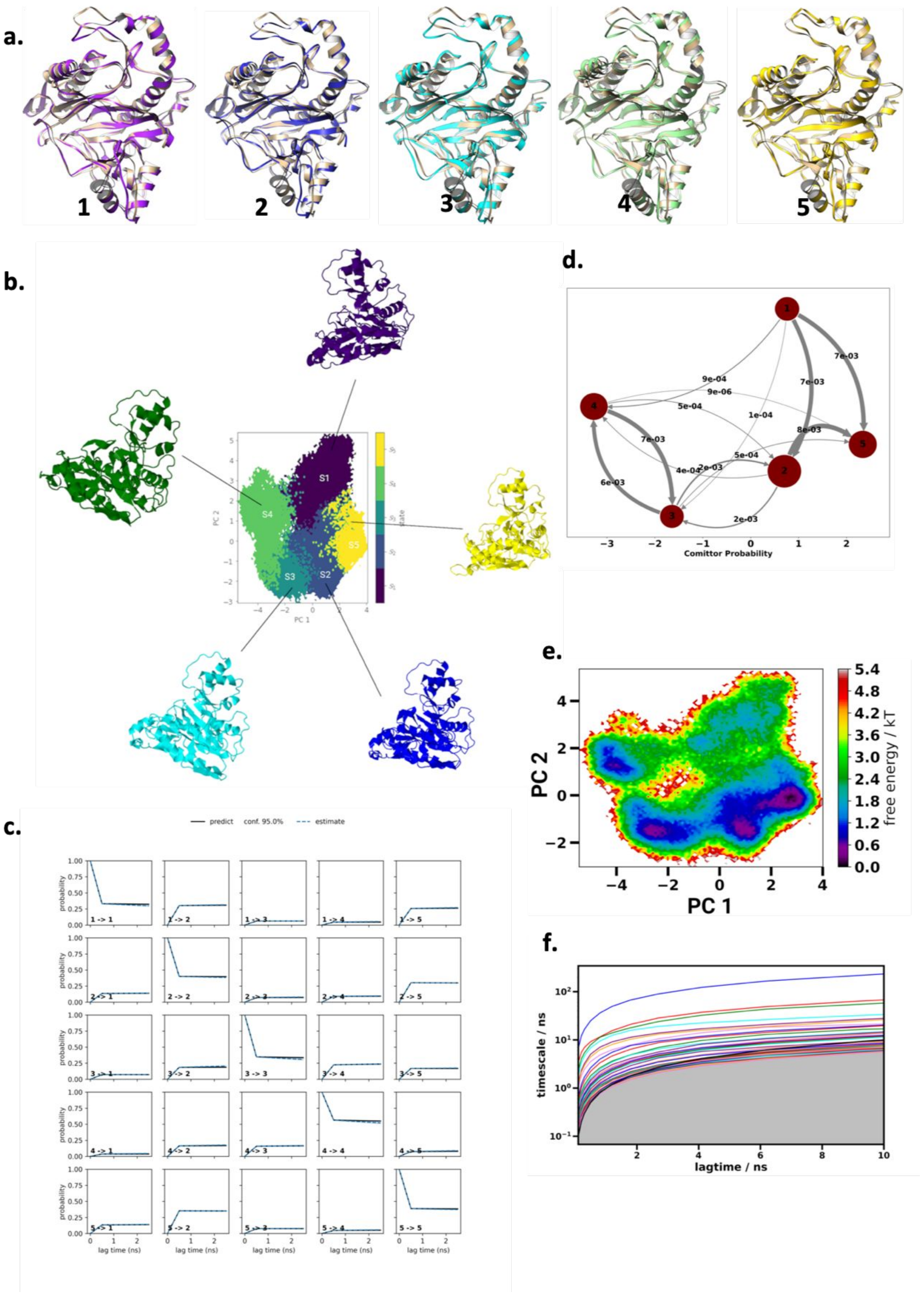

Figure S8: Markov State Model (MSM) of ligand-bound MetAP-II. (a) each metastable state superimposed on the crystal structure 1yw9 (tan); (b) macrostate distribution projected on the first two principal components along with their representatives; (c) CK test plot; (d) Transition path theory analysis; net flux plot shows the probabilities of each transition in the relevant direction per unit time;(e) Free Energy Landscape; (f) Implied timescale plot.

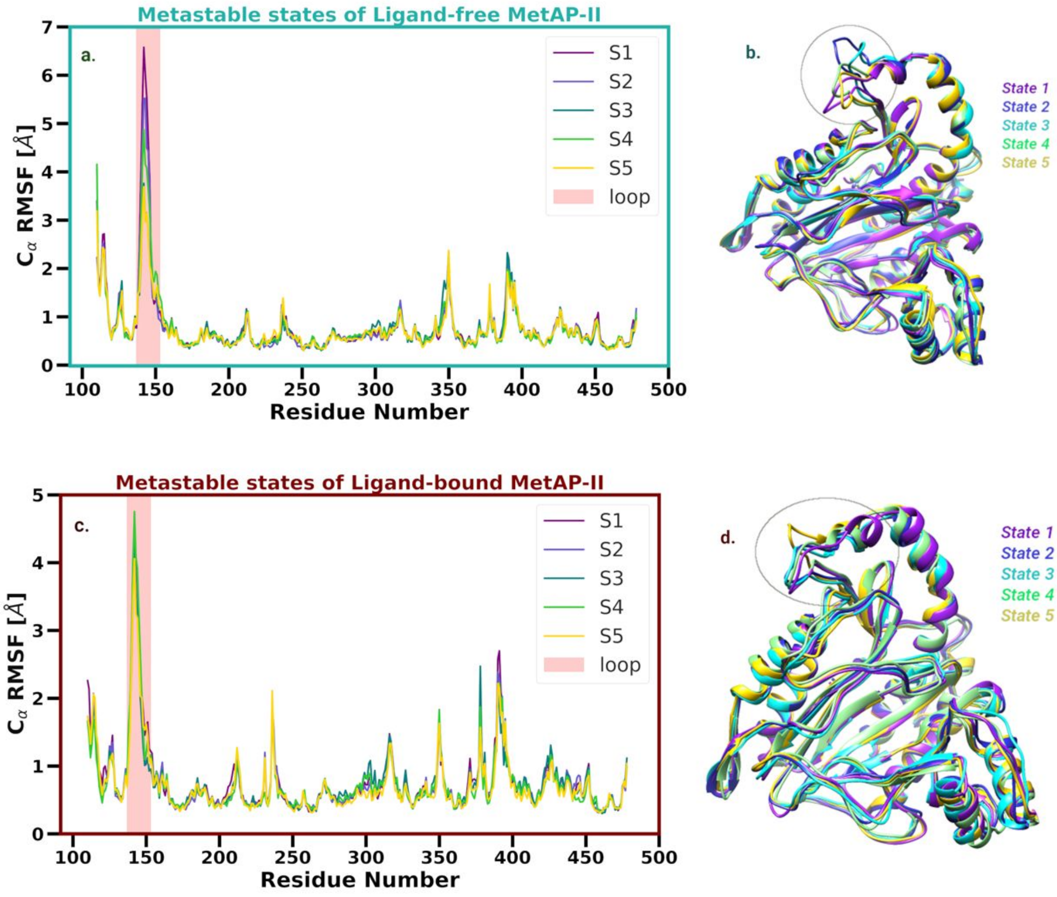

Figure S9: Cα-RMSF of each metastable state in (a) apo MetAP-II and (b) representative conformations obtained from MSM analysis highlighting the flexible loop region; (c) Cα-RMSF of each metastable states in the ligand-bound MetAP-II, and (d) representative conformations obtained from MSM analysis indicated stability of the loop region as compared to apo MetAP-II.

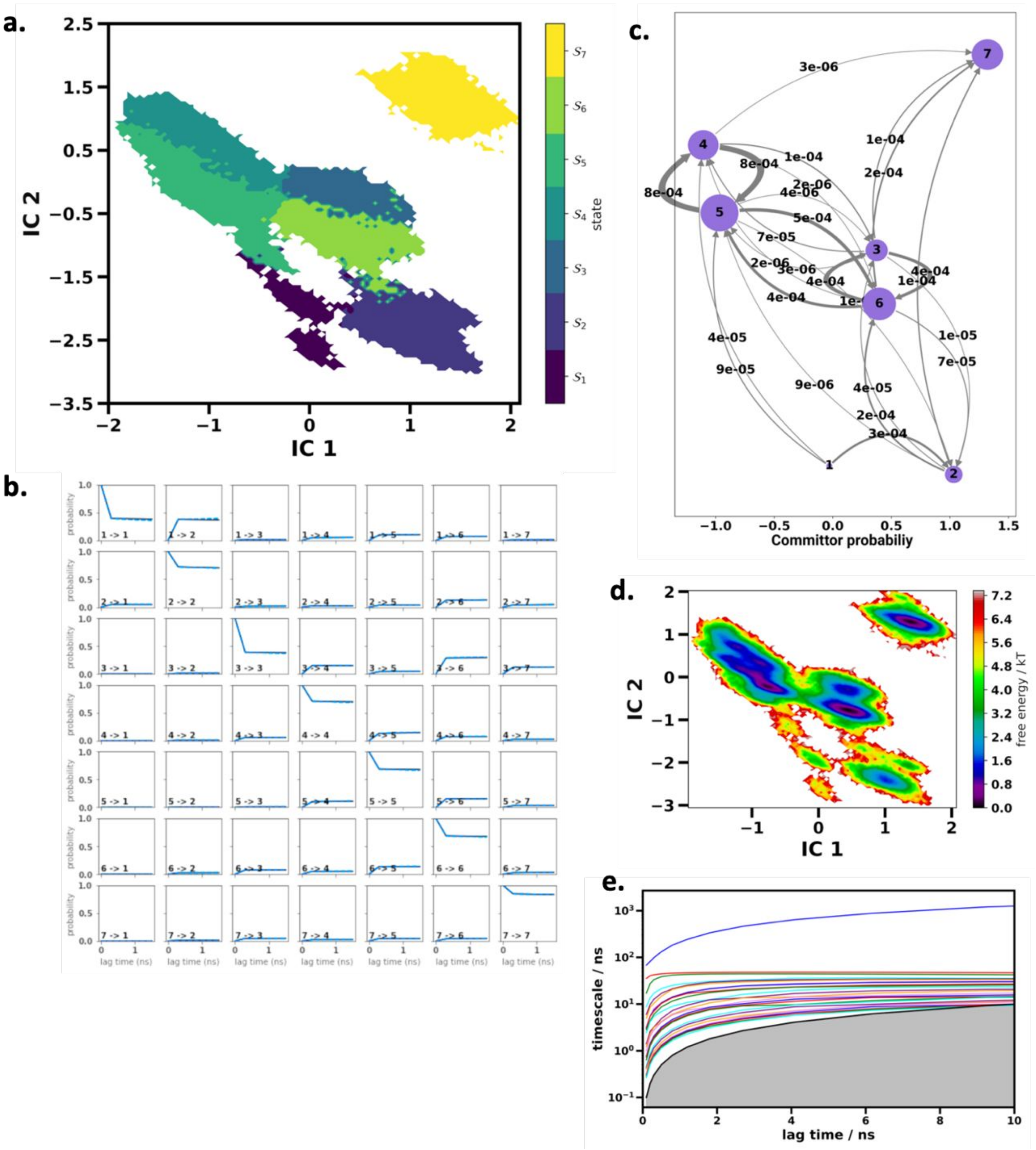

Figure S7: Markov State Model of the combined trajectories of MetAP-II. (a) macrostates distributions, conformations projected on the first two time-lagged independent components; (b) Chapman-Kolmogorov (CK) test plots; (c) Transition path theory analysis, net flux plot shows the probabilities of each transition in the relevant direction per unit time; (d) Free Energy Landscape; (e) Implied timescale plot.

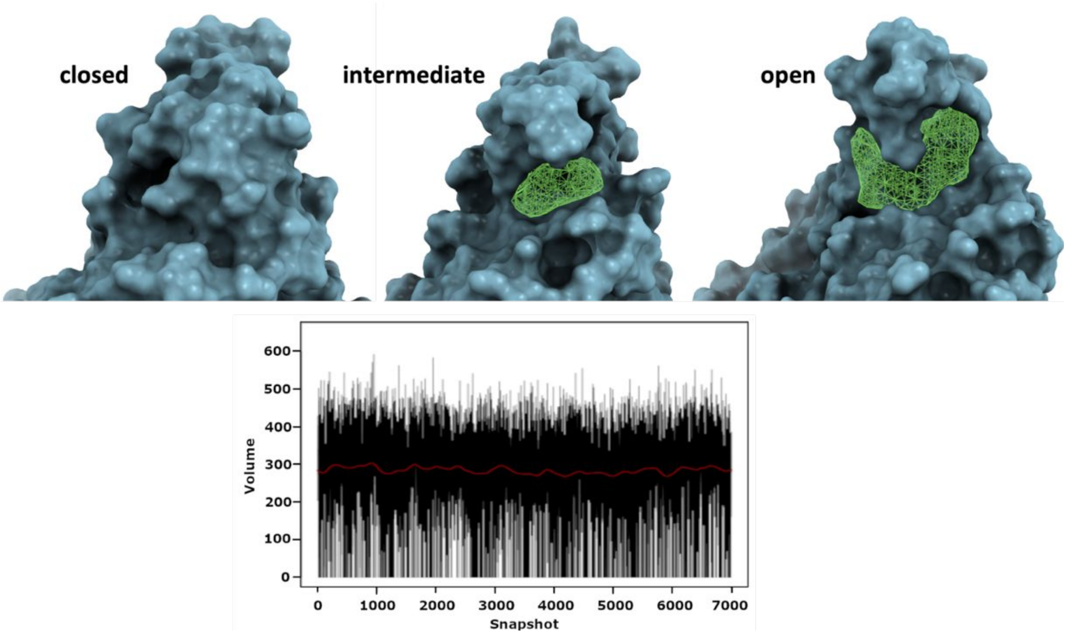

Figure S8: Pockets in the closed, intermediate (279 Å³), and open (405 Å³) conformation of the disordered loop. The pocket is represented as a mesh (green). The running average of the pocket volume of each snapshot retrieved from MSMs has been plotted.

Table S1: Intramolecular interactions in the ligand-bound system (Å).

Intramolecular interaction					
**Intramolecular interactions in the apo form that are lost in the ligand-bound state**			**New intramolecular interaction formed after ligand binding**		
	APO	Ligand-bound		APO	Ligand-bound
T140-R354	2.819 ± 0.248	8.286 ± 1.050	I165-L463	5.187 ± 0.425	4.978 ± 0.431
R144-D294	3.392 ± 0.722	6.839 ± 1.259	Y244-T268	5.041 ± 0.229	3.793 ± 0.357
A147-P138	3.977 ± 0.939	6.396 ± 0.497	D245-T268	5.860 ± 0.317	4.028 ± 0.378
H176-Y478	3.084 ± 0.399	5.642 ± 0.433	L328-V344	5.433 ± 0.338	4.892 ± 0.257
A283-Y311	4.929 ± 0.395	5.064 ± 0.421			
D294-R144	3.392 ± 0.722	8.928 ± 1.401			
R326-N329	4.143 ± 0.828	5.580 ± 0.464			
R354-T140	2.819 ± 0.248	8.286 ± 1.050			
R354-D142	1.648 ± 0.094	8.955 ± 1.139			
M384-F219	3.995 ± 0.782	5.737 ± 0.283			
L413-W419	4.930 ± 0.313	6.043 ± 0.375			
R417-T233	4.904 ± 0.555	5.137 ± 0.297			
E459-Y478	3.057 ± 0.246	5.469 ± 0.258			

Table S2: Betweenness Centrality in the apo (ligand-free) and ligand-bound MetAP-II.

APO		LIGAND-BOUND	
G127	5.8	G127	0.0
G132	5.5	G132	1.9
Q141	1.45	Q141	310
D142	0.33	D142	11
G143	0.0	G143	84.8
T151	4.5	T151	3.7
S185	2.1	S185	5.4
G237	11.4	G237	1.0
T239	0.5	T239	16.9
D294	0.2	D294	5.8
T316	0.7	T316	24.9
G330	4.5	G330	0.08
K342	4.9	K342	0.4
G358	15.0	G358	0.08
E379	3.4	E379	10.5
H391	1.6	H391	10.6
N407	0.04	N407	3.07
A414	0.0	A414	3.5
R422	3.2	R422	0.0
E425	4.11	E425	0.0
S426	11.45	S426	6.3
A431	5.6	A431	1.8
G452	2.4	G452	0.0

Table S3: Mean first passage times between metastable states (per ns) in the apo state.

MFPT	1	2	3	4	5
**1**	0.00	171.34	36.62	19.34	14.22
**2**	70.25	0.00	41.55	19.67	22.92
**3**	142.86	249.09	0.00	19.25	12.56
**4**	131.15	232.75	24.54	0.00	10.71
**5**	133.23	244.67	26.60	19.75	0.00

Table S4: Flux path in the apo state of MetAP-II.

Percentage	Path
52.7	[1 5]
31.7	[1 4 5]
7.9	[1 2 4 3 5]
7.7	[1 2 4 5]

Table S5: Mean first passage times between metastable states (per ns) in the ligand-bound MetAP-II

MFPT	1	2	3	4	5
**1**	0.00	16.85	67.00	82.67	26.53
**2**	48.40	0.00	53.21	73.95	25.35
**3**	73.56	30.91	0.00	27.11	53.76
**4**	74.10	38.13	22.81	0.00	61.23
**5**	43.82	12.78	64.92	86.06	0.00

Table S6: Flux path in the ligand-bound state of MetAP-II.

Percentage	Path
47.5	[1 5]
46.3	[1 2 5]
3.2	[1 4 3 5]
2.3	[1 4 3 2 5]
0.7	[1 4 2 5]

Table S7: Mean first passage times between metastable states (per ns) in the combined trajectories of MetAP-II

MFPT	1	2	3	4	5	6	7
**1**	0.00	210.51	220.23	240.04	156.49	112.11	345.04
**2**	1674.19	0.00	194.82	288.05	201.81	90.40	288.21
**3**	2152.24	665.41	0.00	237.33	165.71	51.35	317.17
**4**	2183.99	772.38	249.44	0.00	44.53	141.37	463.01
**5**	2175.71	759.62	248.28	108.60	0.00	122.73	452.05
**6**	2144.54	659.09	147.76	236.18	147.01	0.00	332.99
**7**	2144.79	627.07	185.19	317.07	234.70	105.18	0.00

Table S8: Consensus clusters from FTMap highlighting the druggability of the identified cryptic pocket

<table>
<tr><th>Selected Structure</th><th># of Consensus clusters</th><th># of fragments in the top 3 consensus clusters</th></tr>
<tr><td rowspan="3">Open Conformation</td><td rowspan="3">08</td><td>21</td></tr>
<tr><td>20</td></tr>
<tr><td>15</td></tr>
<tr><td rowspan="3">Intermediate Conformation</td><td rowspan="3">10</td><td>16</td></tr>
<tr><td>16</td></tr>
<tr><td>16</td></tr>
</table>

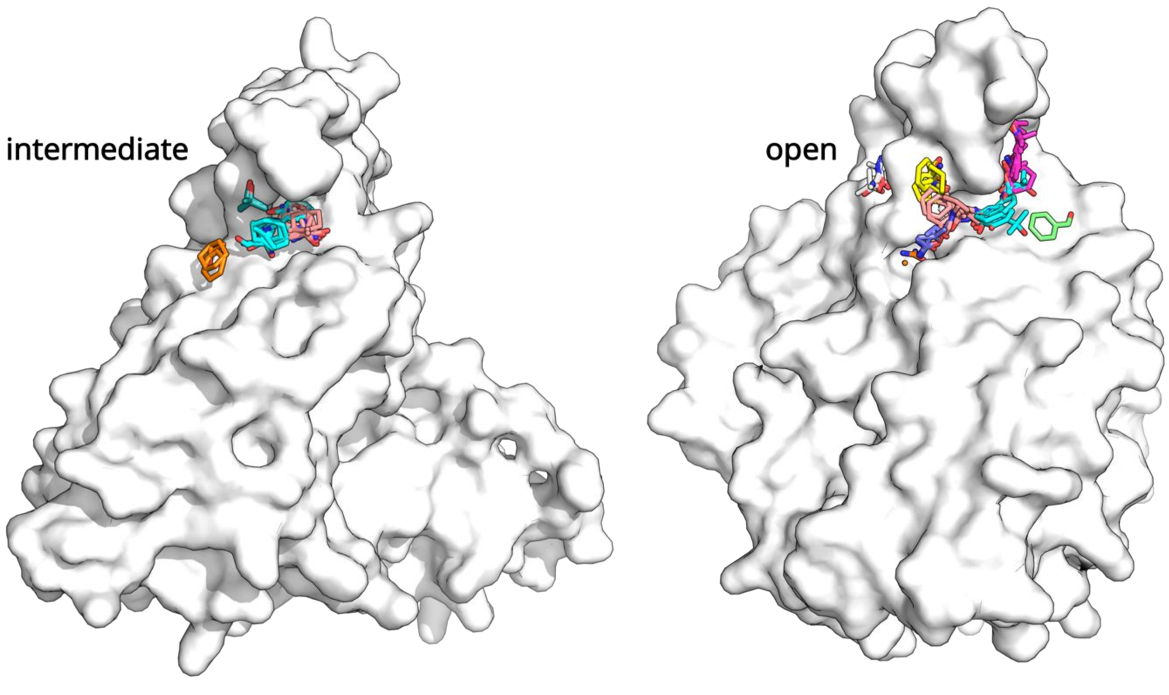

Figure S12: Fragment binding in the intermediate, and open conformation of the disordered loop as determined using FTMap.
